# Institutional design of community energy storage: Value creation, capture, and distribution

**DOI:** 10.1016/j.isci.2026.118075

**Published:** 2026-11-03

**Authors:** Na Li, Binod Koirala

**Affiliations:** 1 Urban Energy Systems Laboratory, Empa, Swiss Federal Laboratories for Materials Science and Technology, Überlandstrasse 129, 8600 Dübendorf, Switzerland

**Keywords:** energy communities, institutional design, community energy storage, market integration, value capture, distributional outcomes, local energy sharing, electricity markets, distributed energy resources

## Abstract

Community energy storage can create economic value, but how ownership, market access, pricing, and network tariffs shape its creation, capture, and distribution alongside grid impacts remains unclear. We develop an integrated modeling framework and apply it to a Swiss energy community case study. Broader market access creates additional storage value, while under dynamic pricing community ownership allows members to retain more of it than third-party ownership. Local sharing prices can redistribute savings but have limited influence when value is driven by market arbitrage. Network tariffs change the relative benefits of individual and shared batteries and, in the community examined, narrow PV-non-PV savings gaps under shared storage. Broader market access can increase peak imports and exports, whereas contracted-capacity charges can moderate import peaks. These findings suggest that institutional design can play an important role in balancing economic performance, distributional outcomes, and grid impacts.

## Highlights

• Market access unlocks additional value from community energy storage
• Storage ownership shapes how much of that value community members retain
• Ownership and network tariffs shape how savings are distributed among households
• Market access can raise grid peaks while capacity charges moderate peak imports

## Graphical abstract

**Figure:**
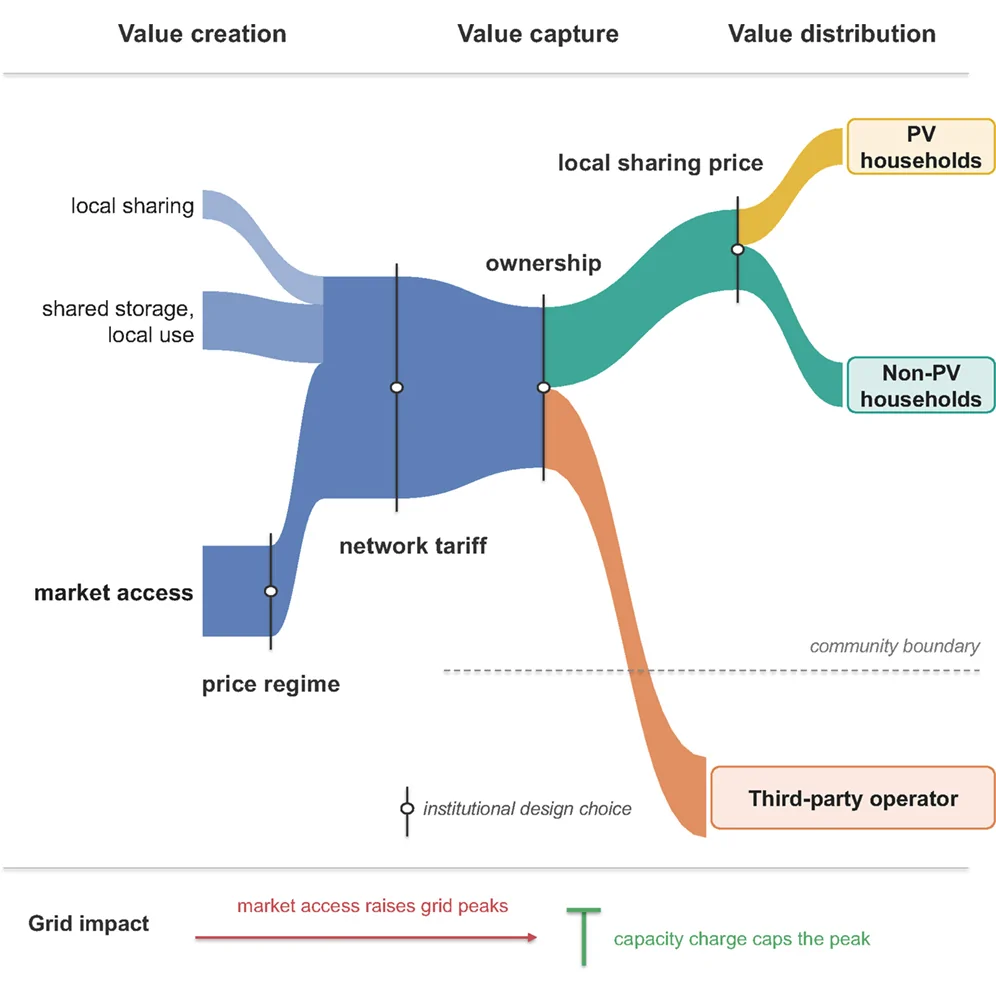

## Teaser

Applied sciences; Energy engineering; Energy storage

## Introduction

The transition toward climate-neutral energy systems is increasingly shifting from a challenge of technology deployment to one of institutional coordination.^1^ Distributed energy resources such as photovoltaic (PV), batteries, electric vehicles, as well as energy management systems, are becoming technically mature and commercially viable. At the same time, policymakers are seeking new ways to integrate these resources into electricity systems while maintaining economic efficiency, grid stability, and social acceptance.^2^^,^^3^^,^^4^^,^^5^ In this context, energy communities have emerged as a promising organizational model for coordinating distributed energy resources at the local level, enabling citizen participation, collective ownership, and local value creation.^6^^,^^7^^,^^8^^,^^9^^,^^10^ Across Europe, regulatory developments, including the introduction of renewable energy communities^11^ and citizen energy communities,^12^ have further expanded the scope of collective ownership and decentralized energy governance.^13^^,^^14^ As these communities scale, institutional choices about who owns the assets,^15^^,^^16^ how they reach markets, and how they price electricity are becoming central to how community energy systems perform.^17^^,^^18^^,^^19^^,^^20^

Among the various energy assets within energy communities, energy storage occupies a particularly important position. By shifting electricity across time, storage can coordinate variable renewable generation with local demand, increase self-consumption, reduce grid dependence, alleviate local network congestion, and provide flexibility to both local and national energy systems.^21^^,^^22^^,^^23^^,^^24^^,^^25^^,^^26^^,^^27^ These capabilities enable storage to create multiple forms of economic and system value, making it a key component of emerging community energy systems. However, technical capabilities do not automatically translate into realized benefits. Whether storage value can be created, who is able to capture that value, and how it is ultimately distributed among community members depend on the institutional arrangements within which it operates.

Research on community energy storage (CES) has developed along several separate trajectories. Some research seeks to maximize the technical and economic performance of storage through improved sizing,^25^^,^^28^^,^^29^^,^^30^^,^^31^^,^^32^ increased self-consumption,^33^^,^^34^ peak shaving,^35^^,^^36^ local energy sharing,^23^^,^^37^^,^^38^^,^^39^^,^^40^^,^^41^^,^^42^ flexibility provision,^35^ market integration,^43^^,^^44^ and operational strategies.^25^^,^^33^^,^^36^^,^^45^^,^^46^^,^^47^ Other work focuses on institutional design, including ownership, governance, business models, and regulatory arrangements.^48^^,^^49^^,^^50^^,^^51^^,^^52^^,^^53^^,^^54^^,^^55^^,^^56^^,^^57^ A further trajectory examines social acceptance, fairness, justice, participation, and the inclusion of vulnerable or underrepresented groups.^58^^,^^59^^,^^60^^,^^61^^,^^62^^,^^63^^,^^64^^,^^65^ As a result, CES research remains fragmented: techno-economic models often provide a constrained and institutionally static representation of storage value by treating ownership, pricing, market access, network tariffs, and allocation rules as fixed assumptions, while governance- and justice-oriented studies identify these institutional dimensions as critical but rarely translate them into quantifiable design variables. Consequently, the literature lacks an integrative framework that explains how institutional arrangements shape both the magnitude of storage value and its distribution among community actors.

This gap is becoming more important because the nature of storage value is evolving. In early energy community applications, energy storage was primarily deployed for local coordination to increase self-consumption and reduce electricity expenditures. Today, with increasing market liberalization, storage is increasingly becoming a market-facing asset, capable of participating in electricity procurement, local trading, flexibility services, balancing markets, and virtual power plant platforms.^24^^,^^44^^,^^66^^,^^67^^,^^68^^,^^69^^,^^70^ However, access to these value streams alone does not guarantee meaningful economic benefits. The profitability of market participation depends not only on the ability to access external markets but also on the price signals under which storage operates. Flat electricity tariffs provide limited opportunities for arbitrage and temporal value shifting, whereas time-varying and dynamic pricing schemes can substantially expand the economic value available to storage assets through temporal price spread. Consequently, value creation in CES depends jointly on market access, the pricing environment, and the network tariff that determines what the community pays for the grid it uses.

The capture and distribution of storage value are shaped by ownership and cost allocation. Ownership determines who invests in the battery, who controls its operation, and who has the first claim on the resulting revenues. The same battery owned by an individual household, by the community collectively, or by a third-party operator places investment risk, operational control, and revenue in very different hands.^54^^,^^55^^,^^71^ These configurations may employ similar technologies, but they represent distinct institutional arrangements and may therefore produce substantially different economic and social outcomes even under the same market and price conditions.^72^ Moreover, energy communities are rarely uniform: some members own rooftop PV and others do not; some provide capital, while others mainly consume electricity. Under such conditions, the arrangement that maximizes total community value does not necessarily distribute that value evenly or fairly across members.^73^ Ownership, therefore, matters not only because it allocates investment and operational authority, but because it determines who bears risk and who receives revenues.

Taken together, these developments reveal a fundamental shift in the role of storage within energy communities. The central question is no longer whether storage creates value, but rather under what institutional conditions value can be created, who captures that value, and how it is distributed among stakeholders. Yet current knowledge remains fragmented. Existing studies have examined ownership structures, market participation mechanisms, and benefit-sharing arrangements largely in isolation. As a result, we still lack a coherent understanding of how institutional design determines storage value. In particular, it remains unclear how ownership structures, market participation regimes, pricing environments, and network tariff design interact to shape not only how much value storage creates, but also who captures that value and how it is ultimately distributed among stakeholders.

To address this gap, this study develops an integrated modeling framework for analyzing storage-enabled energy communities across four institutional dimensions: ownership structure, market integration, retail price regime, and network tariff design. Ownership structures determine who invests in and controls storage assets; market integration regimes determine which value streams can be accessed; retail price regimes determine the economic incentives available for storage operation; and network tariffs shape the cost of grid use and therefore the incentives for managing imports, exports, and peak demand. In particular, whether network charges are based primarily on energy consumption or on network capacity can influence how storage responds to both market and grid conditions.^74^^,^^75^ By systematically comparing alternative institutional configurations, the framework enables an integrated assessment of how storage value is created, captured, and distributed among community members and other actors. The results provide insights for policymakers, regulators, investors, community organizers, and market participants seeking to design economically viable and socially sustainable energy communities in increasingly distributed electricity systems.

The institutional setting examined here is the Swiss local electricity community (*lokale Elektrizitätsgemeinschaft*, LEG), which became possible on January 1, 2026, under the revised Electricity Supply Ordinance.^76^ Participants retain their individual grid connections and metering points and exchange locally generated electricity over the public distribution grid. The price at which this electricity is settled among members is agreed within the community, and electricity generated and consumed within the community receives a statutory reduction of the network usage tariff of 40% where no voltage transformation is required and 20% otherwise; electricity supplied by a storage facility qualifies only up to the amount the storage has drawn from the community. These two provisions, an internally set sharing price and a network-charge reduction tied to local supply, are the channels through which the institutional arrangements examined here create and distribute value, and both are represented explicitly in the model.

## Results

Before presenting the comparative results, Figure 1 summarizes the heterogeneity in annual electricity consumption and PV generation across the seven units used in the case study, together with the seasonal profiles of community demand and PV generation (Table 1). Figure 2 provides an overview of the model configurations and institutional settings evaluated, ranging from baseline without an energy community to individual, community-owned, and third-party-owned storage under different market access and network tariff designs (Table 2).

**Figure 1:**
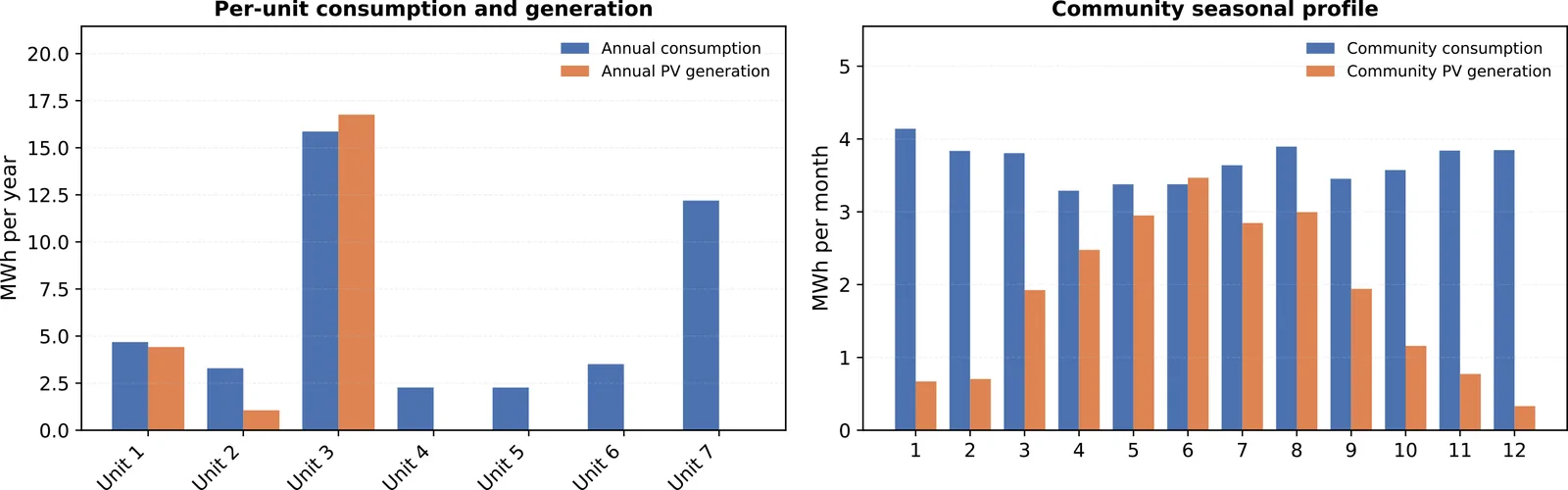
Measured electricity profiles defining the NEST case study Left: annual consumption and PV generation of the seven included units. Right: aggregate monthly consumption and generation. The variation in demand and PV ownership provides the heterogeneity on which the institutional arrangements examined here operate.

**Table 1:** The seven NEST units included in the case study over calendar year 2025

Unit	Consumption (MWh/yr)	PV generation (MWh/yr)	Peak demand (kW)	Load factor	PV/consumption
1	4.68	4.42	3.64	0.15	0.94
2	3.29	1.06	1.90	0.20	0.32
3	15.86	16.76	7.55	0.24	1.06
4	2.27	–	1.42	0.18	–
5	2.26	–	2.69	0.10	–
6	3.51	–	2.57	0.16	–
7	12.20	–	3.78	0.37	–
Community	44.08	22.24	12.10	0.42	0.50

Consumption and generation are measured at hourly resolution. Units are numbered sequentially for presentation; three of the ten NEST units are excluded because their records are incomplete. The load factor is annual consumption divided by the product of peak demand and the hours in the year.

**Figure 2:**
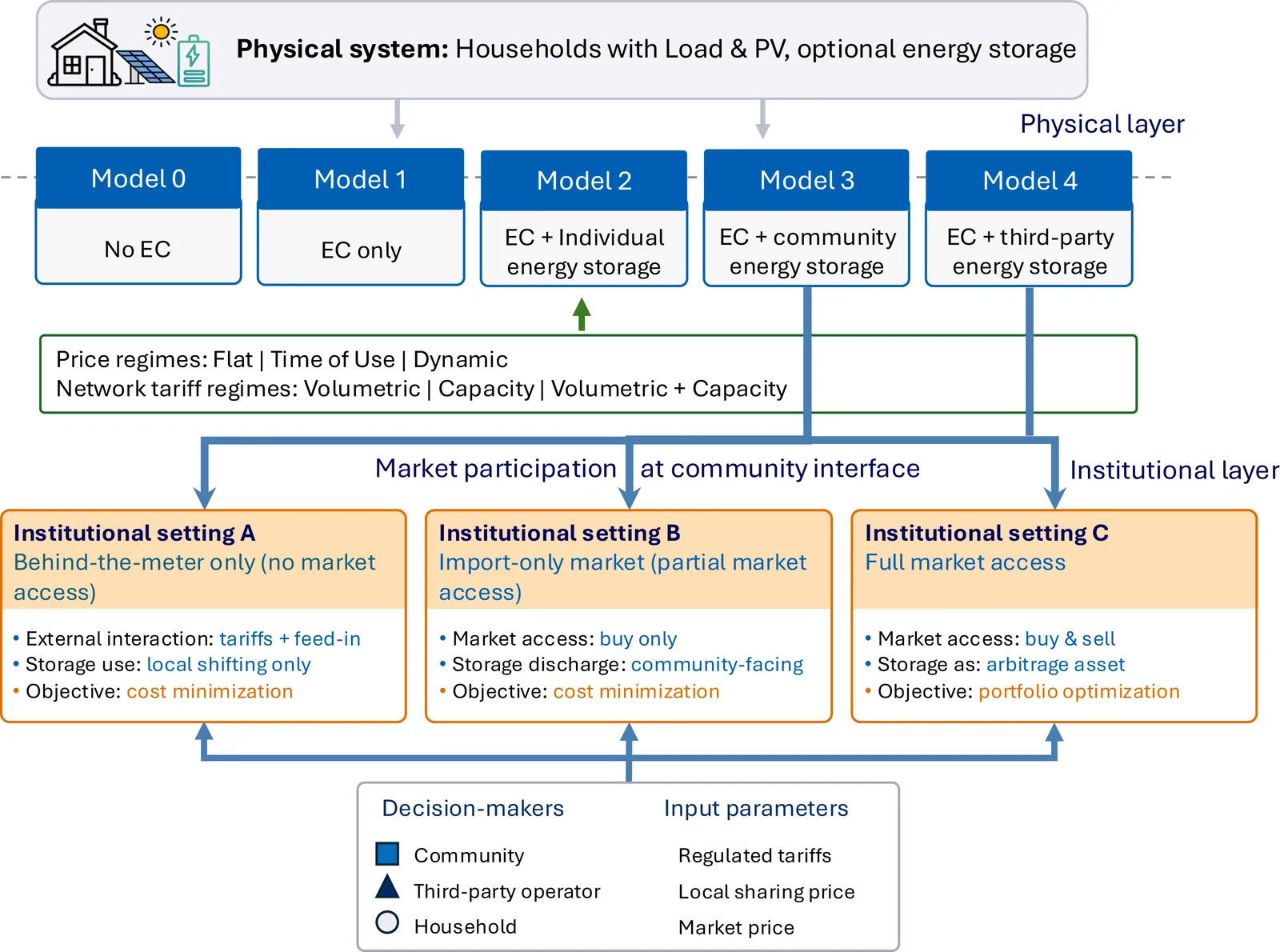
Conceptual framework for evaluating institutional design in community energy systems The framework integrates physical system operation, institutional configurations, network tariff design, and economic settlement within a single structure. Households are characterized by electricity demand, PV generation, and optional storage. Institutional design is defined along four dimensions: ownership structure (models 0–4), determining control and value allocation of shared assets; market integration (settings A–C), governing interaction with external electricity markets; the retail price regime, governing the incentive available for storage operation; and the network tariff archetype (T1–T3, with T0 as an energy-only benchmark), governing how network use is charged. Physical dispatch is driven by system-level optimization under external price signals and tariff constraints, while internal economic settlement is determined ex post. This separation enables independent assessment of efficiency, distributional outcomes, grid impacts, and third-party incentives.

**Table 2:** Institutional design space of community energy models

	Setting A (no market access)	Setting B (partial market access)	Setting C (full market access)
No storage	M0/M1	–	–
Individual storage	M2	–	–
Community-owned CES	M3A	M3B	M3C
Third-party CES	M4A	M4B	M4C

Ownership structure (rows) and market integration settings (columns) define the modeling configurations. Each configuration is evaluated under the energy-only benchmark (T0), three network tariff archetypes (T1–T3), and three electricity price regimes, except that the market access settings (B and C) are evaluated under dynamic pricing only, yielding 76 simulation scenarios.

### Storage extends the value of local sharing, but network tariffs shape the relative advantage of individual and shared batteries

Local energy exchange provides modest community cost savings across network tariff structures and retail price regimes (Figure 3). Its benefit is limited by the temporal mismatch between PV generation and household demand: surplus electricity can be shared directly only when generation and consumption coincide. Storage extends this value by allowing locally generated electricity to be used at a later time.

**Figure 3:**
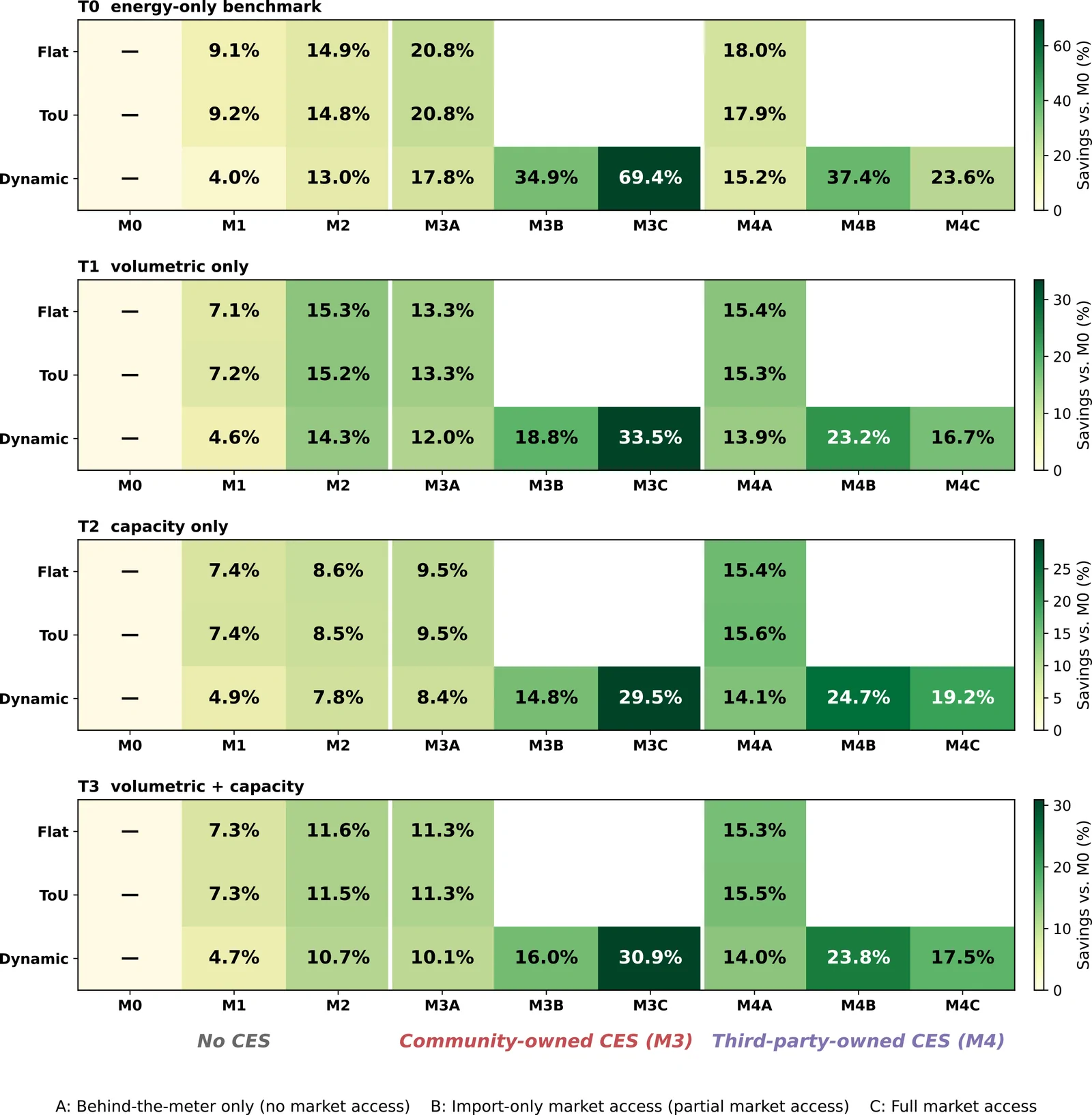
Community cost savings across institutional configurations, price regimes, and network tariff archetypes Savings (Equation 2) are shown for each institutional configuration and retail energy price regime under the energy-only benchmark and the three network tariff archetypes. T0 is the energy-only benchmark, which excludes network charges to isolate the energy-related value of each configuration. T1, T2, and T3 represent volumetric, contracted-capacity, and combined volumetric-capacity network tariffs, respectively (Tables 3^4^, and 5). Configuration labels are used consistently throughout the paper (Table 2): M0 is the no-community reference; M1 represents local energy exchange without storage; M2 energy community with individual-owned storage; and M3 and M4 shared storage owned by the community and by a third-party operator, respectively. The suffixes A, B, and C denote behind-the-meter operation, import-only market access, and full market access. Setting A is evaluated under all three retail price regimes; settings B and C are shown only under dynamic pricing, where external market participation creates an additional intertemporal value stream. Savings are reported at the CV-minimizing local sharing price *π*^loc^, used here as a benchmark for distributional evenness across households. For M1–M3, total community cost is invariant to *π*^loc^ because local settlement is zero-sum within the community, whereas for M4 it varies with *π*^loc^ because service payments cross the community boundary. The CV-minimizing *π*^loc^ is defined as the sharing price that minimizes the CV of relative household savings, Equation 1.

**Table 3:** Energy component of the retail electricity price, excluding value-added tax

Season	High tariff (Rp/kWh)	Low tariff (Rp/kWh)
Winter	12.70	12.20
Summer	12.40	11.90

Glattwerk AG.^83^

**Table 4:** Network tariff archetypes used in the analysis

Archetype	Charging basis	Main economic signal
T0	energy-only benchmark (no network charge)	grid exchange is unpriced
T1	electricity withdrawn	prices energy exchanged with the grid
T2	contracted connection capacity	prices the power capacity available at the connection
T3	electricity withdrawn and contracted capacity	combines energy- and capacity-based signals

T1–T3 are calibrated *ex ante* to recover the same network revenue from model 0.

**Table 5:** Network tariff parameters used in the analysis, excluding VAT

Component	Basis	T0	T1	T2	T3
Volumetric charge	Rp/kWh, high tariff	–	8.10	–	3.490
	Rp/kWh, low tariff	–	7.10	–	3.211
Capacity charge	CHF/kW/year, contracted	–	–	21.86	12.18
Fixed charge	CHF/month/connection	–	7.50	7.50	7.50
Metering charge	CHF/month/connection	–	5.00	5.00	5.00
Model 0 network revenue (CHF/year)		0	3697.6	3697.6	3697.6

T1 uses the published household rates at the case study location. T2 and T3 are calibrated to the same model 0 network revenue.

Storage increases community savings, but the relative advantage of individual and shared batteries depends on network tariff design. We first compare their energy-related costs alone (T0), isolating the value of storage from network charges. Under this benchmark, community-owned shared storage restricted to internal balancing (M3A) outperforms individual behind-the-meter batteries (M2) across all retail price regimes, reflecting the benefits of pooling generation and demand across households. When network charges are included, this relationship changes: individual batteries perform better under the volumetric tariff (T1), shared storage retains a slight advantage under the contracted-capacity tariff (T2), and the two perform similarly under the combined tariff (T3). The economic advantage of shared storage therefore depends not only on its physical pooling capability, but also on how network costs are recovered.

This difference arises from how network charges are applied at individual metering points. Behind-the-meter batteries reduce household grid withdrawals and directly lower volumetric network charges. By contrast, electricity discharged from shared storage remains subject to network charges at the receiving household’s meter, while the storage requires a separate connection. These costs can offset the benefits of pooling generation and demand across households. Under contracted-capacity charging, reducing metered energy withdrawal does not reduce the household’s connection charge (household connection ratings are held fixed), weakening the network-side advantage of individual batteries. Shared storage can also shift locally generated electricity across time while preserving its eligibility for the statutory network charge reduction (Article 19h of the Swiss Electricity Supply Ordinance [StromVV]^76^).

The comparison further highlights the importance of accounting for network costs when evaluating community storage. The energy-only benchmark isolates the economic value associated with electricity purchases, exports, and storage operation, whereas the charged network tariff archetypes account for the additional costs of using the distribution network. Under dynamic pricing, community-owned storage with full market access (M3C) remains the highest saving configuration across all three network tariff archetypes (29.5%–33.5% under T1–T3). Network tariff design therefore affects the economic value of storage and the relative advantage of individual versus shared batteries without overturning the broader institutional pattern. The following section examines how market access and ownership determine which actors capture the additional storage value.

### Market access unlocks additional storage value, while ownership determines who captures it

We focus on dynamic pricing, under which temporal price variation creates additional opportunities for market participation. Moving from internal balancing (setting A) to import-only access (setting B) and full market access (setting C) progressively expands the activities available to shared storage (Figure 4).

**Figure 4:**
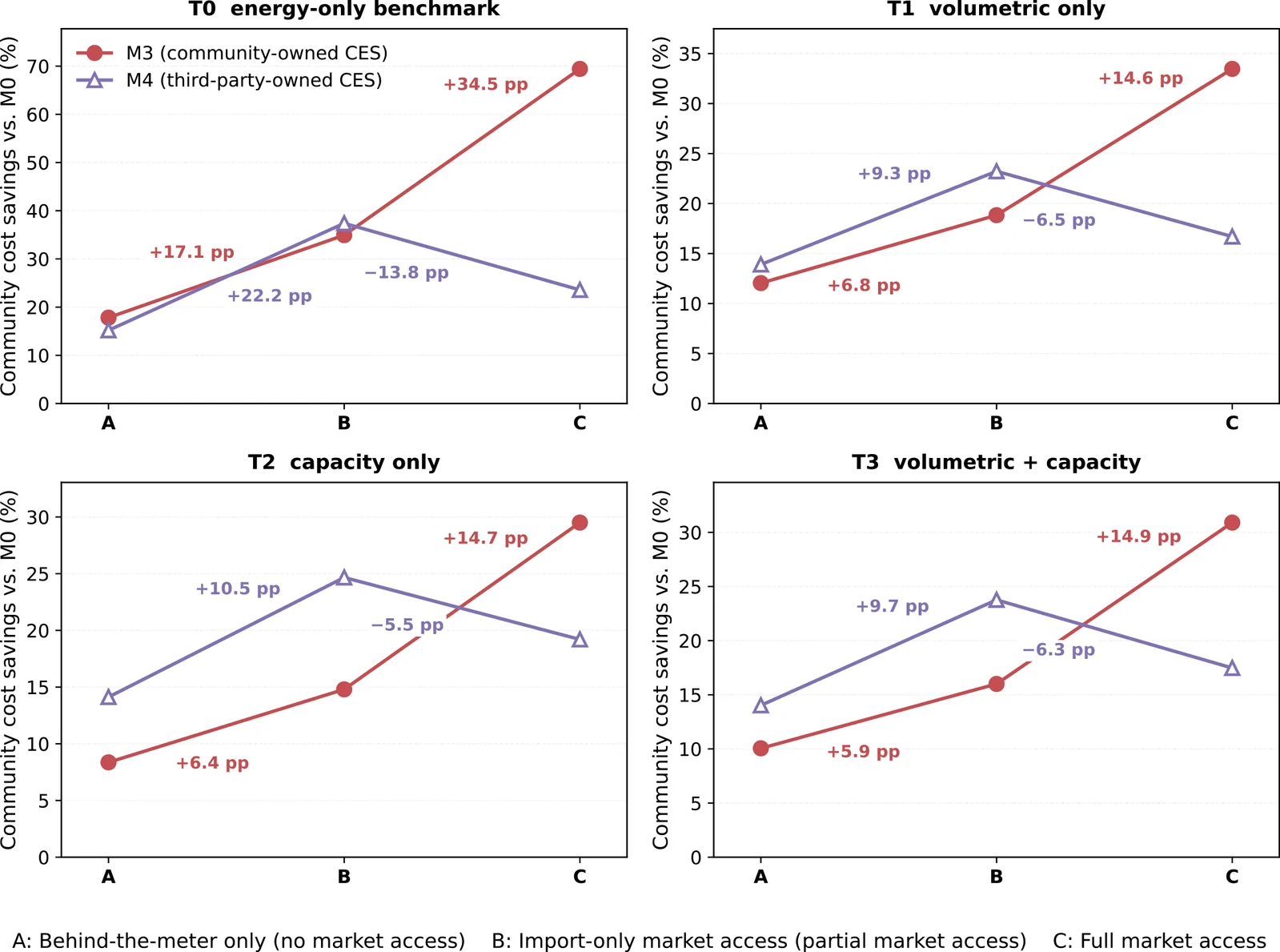
Market access unlocks additional storage value, while ownership determines who captures it Community cost savings across market access settings for community-owned (M3) and third-party-owned (M4) shared storage under dynamic pricing, for the energy-only benchmark (T0) and the three network tariff archetypes (T1–T3). Configuration and tariff definitions follow Figure 3. Savings are measured relative to M0 under the same tariff. Labels indicate changes in community savings between successive market access settings, in percentage points.

Under community ownership, broader market access consistently increases community savings across all netowork tariff archetypes. Full market access creates substantially more value than internal balancing alone, demonstrating that local energy use captures only part of the economic potential of shared storage. Under third-party ownership, however, community savings increase from setting A to B but decline from B to C under every tariff case. Expanding the storage’s market opportunities therefore does not necessarily increase the value retained by community members. This difference can be explained by the financial position of the third-party operator (Figure 5). The operator receives storage service revenue and any positive market arbitrage margin, but also bears arbitrage losses and the network charge on the storage connection. These revenues and costs accrue to the community under M3 and to the external operator under M4. The fixed participation fee is set to zero in the baseline analysis to isolate flow-based revenues. A positive fee would increase the third-party operator’s revenue by the amount collected and reduce community members’ savings by the same amount; an equal per-member fee would fall most heavily on low-consumption households, whose flow-based benefits are smallest.

**Figure 5:**
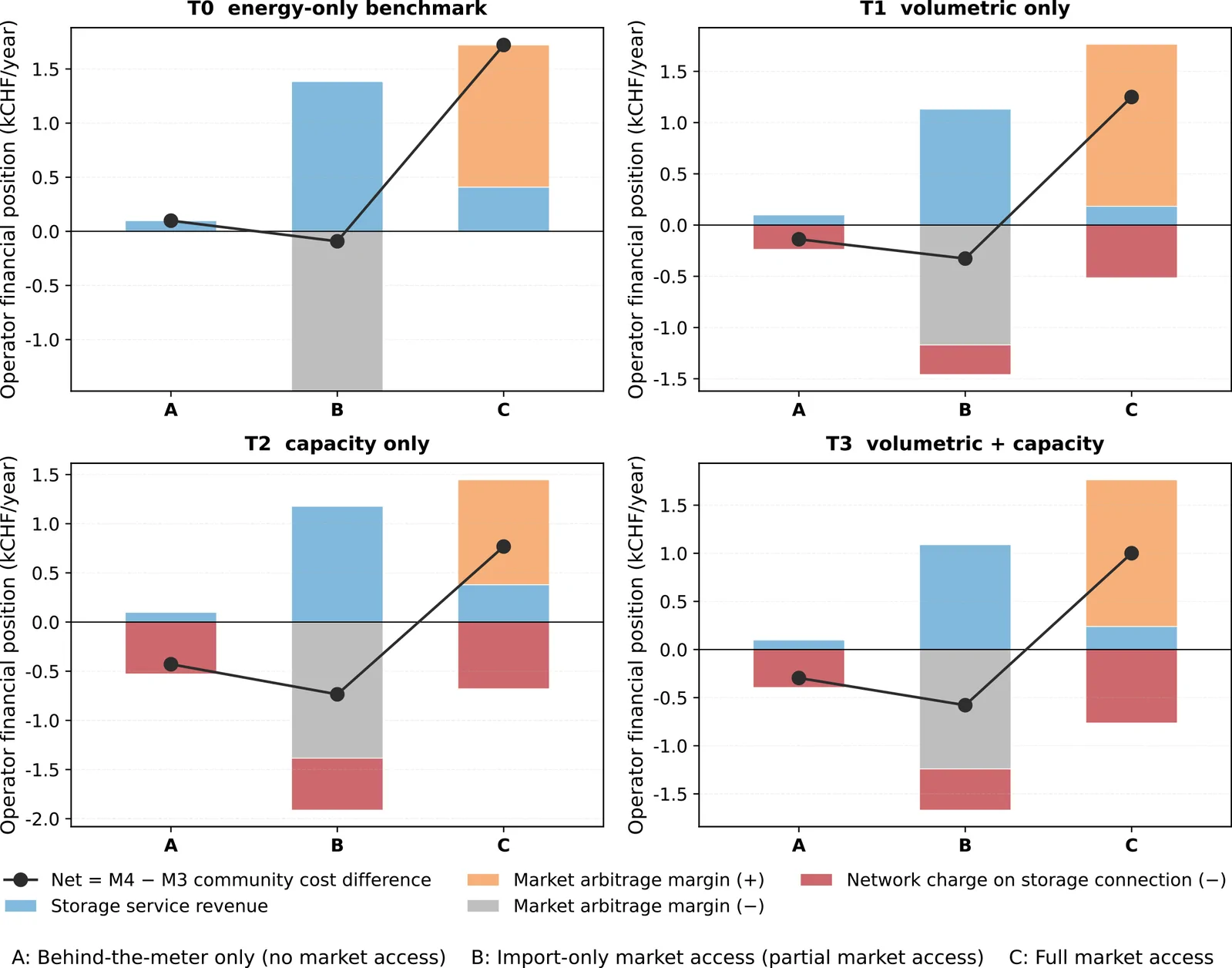
Financial position of the third-party storage operator under dynamic pricing Decomposition of the operator’s financial position under dynamic pricing, for the energy-only benchmark (T0) and the three network tariff archetypes (T1–T3). Positive components comprise storage service revenue and positive market arbitrage margins; negative components comprise arbitrage losses and network charges on the storage connection. The fixed participation fee is zero in the baseline analysis. The black line represents the community cost difference *C*^M4^ − *C*^M3^ under the same market access setting. Positive values indicate lower community costs under community ownership (M3), whereas negative values indicate lower community costs under third-party ownership (M4).

Without external market access, the ownership difference mainly reflects who receives storage service payments and who bears the storage connection charge. Under the energy-only benchmark (T0), community ownership results in lower community costs because third-party ownership introduces a service payment without transferring any network charge. Under T1–T3, the operator bears the storage connection charge, which exceeds its service revenue. Third-party ownership therefore results in lower community costs, despite identical physical storage operation. This reflects the common dispatch objective: the operator is assumed to operate the storage to minimize community cost rather than its own profit and does not adjust dispatch or fees to avoid this loss.

Under import-only market access, the operator’s market arbitrage margin is negative: the storage purchases grid electricity that is later supplied to households, and the margin is calculated on grid transactions only, whereas revenue from local supply enters as storage service revenue. This negative margin, together with any connection charge, outweighs the service revenue; the operator’s position excludes the fixed participation fee, which is zero in the baseline. Third-party ownership consequently transfers these costs outside the community, increasing community savings relative to community ownership. Full market access reverses this outcome: the operator earns a substantial positive arbitrage margin, which is retained by the community under M3 but captured externally under M4.

The comparison shows that third-party ownership transfers both the costs and the benefits of market participation across the community boundary. Network tariffs affect the size of this transfer through the storage connection charge, but the contrasting responses to market access remain consistent across the network tariff archetypes. Market access determines which value streams storage can reach, while ownership determines who ultimately captures their benefits and bears their costs.

### Institutional arrangements and network tariffs shape the distribution of household savings

We evaluate distributional outcomes using the coefficient of variation (CV) (Equation 1) of relative household savings and the difference in mean savings between PV and non-PV households (Figures 6 and 7). A lower CV indicates a more even distribution of relative savings, while the group comparison identifies which households benefit more. For each configuration, the CV-minimizing local sharing price provides a common benchmark for comparing distributional outcomes. In most configurations, this price lies at the lower bound of the admissible range, that is, at the feed-in remuneration, where PV households are indifferent between sharing and exporting.

**Figure 6:**
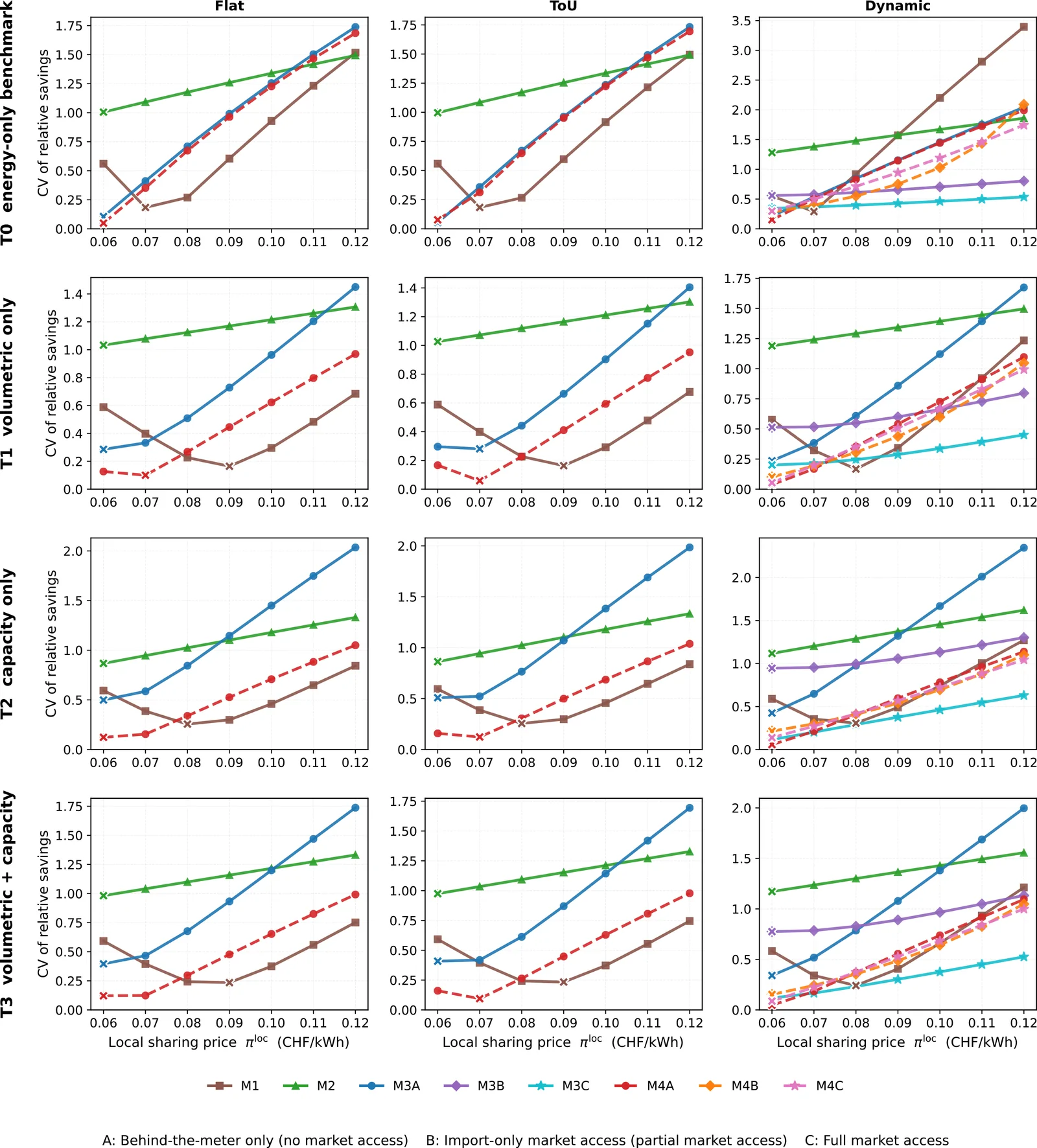
The local sharing price shapes the evenness of household savings only where value passes through local exchange CV of relative household savings as a function of the local sharing price *π*^loc^ across institutional configurations, the energy-only benchmark and network tariff archetypes (rows), and retail price regimes (columns). Lower values indicate a more even distribution of relative savings. Configuration and tariff definitions follow Figure 3. Settings B and C are evaluated under dynamic pricing only.

**Figure 7:**
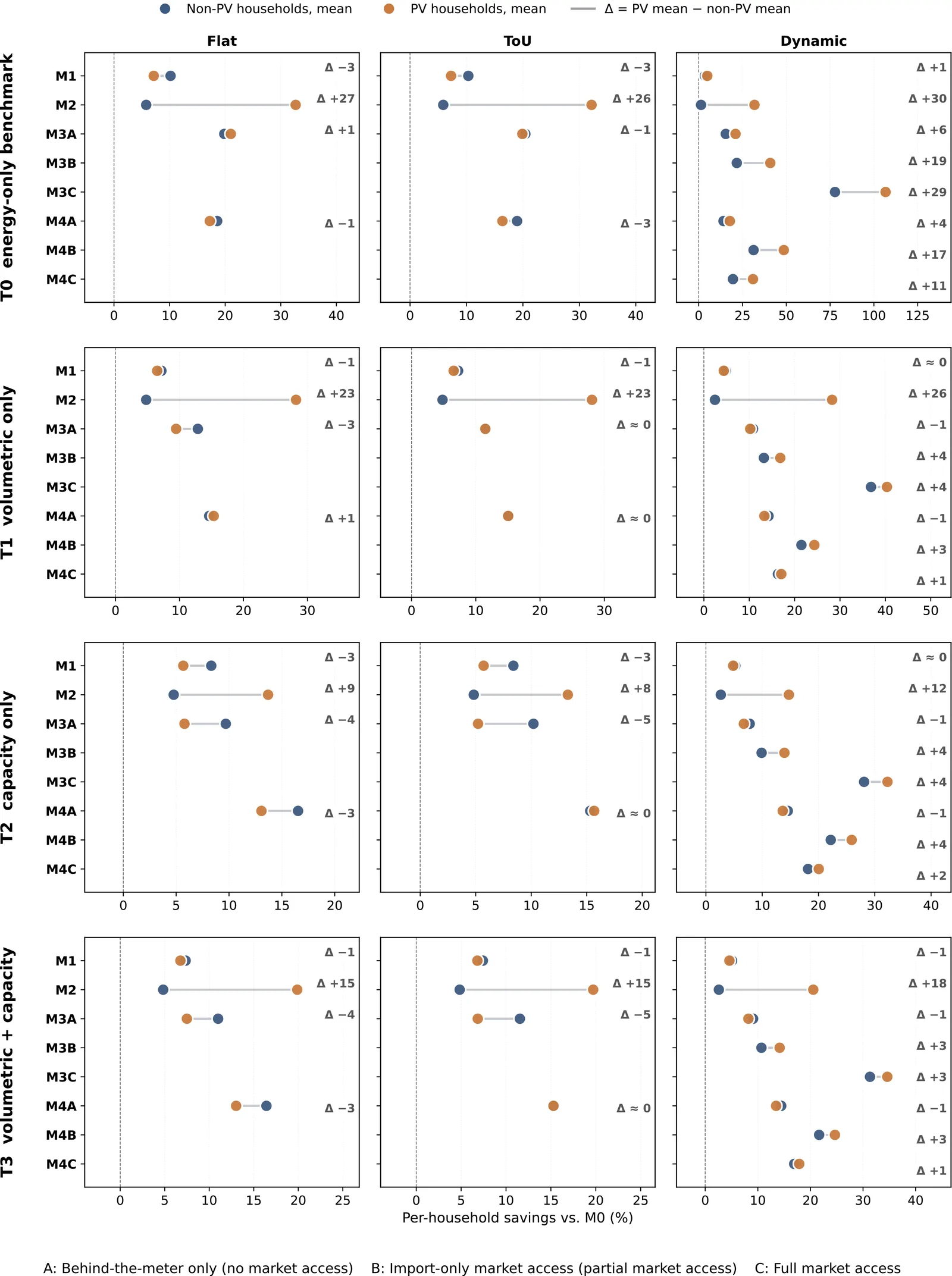
Institutional arrangements and network tariffs shape the distribution of household savings Mean relative cost savings of PV and non-PV households across institutional configurations, the energy-only benchmark and network tariff archetypes (rows), and retail price regimes (columns), evaluated at the CV-minimizing local sharing price. Savings are measured relative to M0 under the same network tariff. The difference $\Delta ={\bar{S}}_{\text{PV}}-{\bar{S}}_{\text{non}-\text{PV}}$ is reported in percentage points; positive values indicate higher mean savings for PV households. Configuration and tariff definitions follow Figure 3.

The effect of the local sharing price depends strongly on the institutional configuration (Figure 6). Local exchange without storage (M1) exhibits an interior minimum in the CV, reflecting how the sharing price divides benefits between producing and consuming households. Individual behind-the-meter storage (M2), by contrast, maintains a relatively high CV even at its minimizing price because storage benefits remain concentrated among households with PV. Shared-storage configurations exhibit different responses depending on ownership and market access, with some showing substantial changes as the sharing price increases. These patterns are broadly preserved across retail price regimes, although network tariff design changes the magnitude of the differences between configurations.

The group comparison further shows how network tariff design affects the distribution of benefits (Figure 7). Individual behind-the-meter storage (M2) consistently favors PV households, as both generation and storage benefits remain concentrated within these households. Shared storage generally produces smaller group differences, although this depends on the tariff structure. Under the energy-only benchmark (T0), market integrated shared storage can also strongly favor PV households. With network charges and the associated statutory reduction, these differences become substantially smaller. For example, the PV-non-PV savings gap under M3C decreases from approximately 29 percentage points in T0 to 3–4 percentage points under T1–T3.

The statutory reduction also affects the total value of local energy use. Under Swiss regulation, electricity generated and consumed within a local energy community qualifies for a reduction in the network usage tariff. The baseline reduction is 40% for the case study community (Article 19h of the StromVV^76^). We vary this rate to examine its effect on community savings (Figure 8).

**Figure 8:**
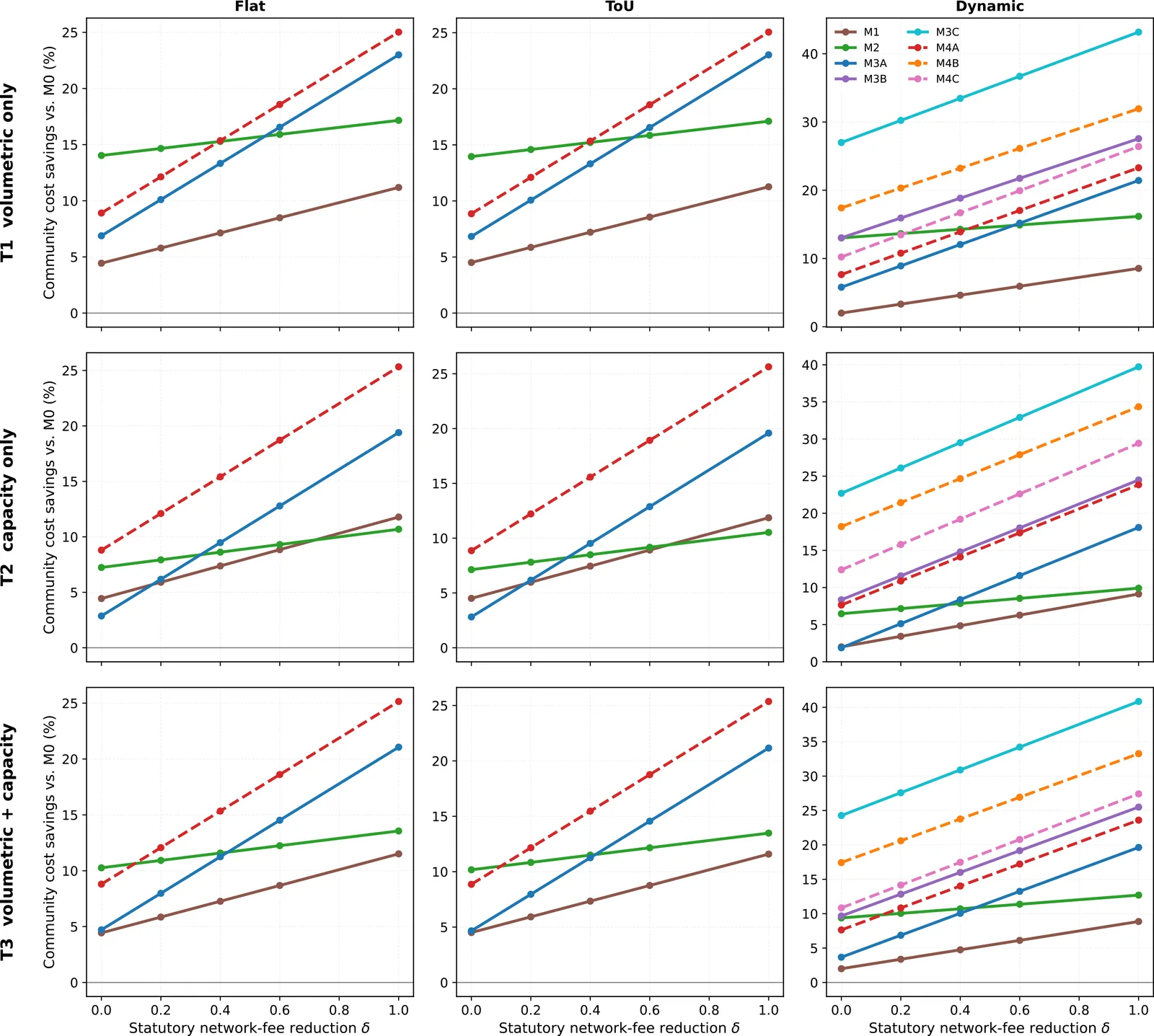
Sensitivity of community cost savings to the statutory network charge reduction Community cost savings as a function of the reduction rate *δ* across institutional configurations, network tariff archetypes, and retail price regimes. The baseline reduction rate is 0.40. Settings B and C are evaluated under dynamic pricing only. Configuration and tariff definitions follow Figure 3. Storage dispatch is held at the schedule optimized for the baseline reduction rate to isolate the direct effect of changing *δ*.

Increasing the reduction rate raises community savings, with the magnitude of the increase depending on the amount of eligible local electricity supplied. Shared storage can increase this amount by shifting locally generated electricity across time, whereas grid-charged electricity does not qualify for the reduction. The sensitivity analysis therefore confirms that the benefit of the reduction depends on the energy flows created by each institutional configuration.

Together, these results show that household-level distributional outcomes depend on asset ownership, market access, local pricing, and network tariff rules. The local sharing price can adjust how value is distributed through local settlement, but it cannot eliminate differences arising from asset ownership. Network tariff design and statutory reductions further affect the economic benefits associated with local energy use, while the main institutional patterns remain consistent across the examined regulatory settings.

### Market-integrated storage amplifies grid interaction, creating a trade-off between community savings and grid peaks

Market access increases the economic value of shared storage but also changes how the community interacts with the upstream grid. Figure 9 compares the load-duration curves of community grid exchange across institutional configurations and network tariff archetypes.

**Figure 9:**
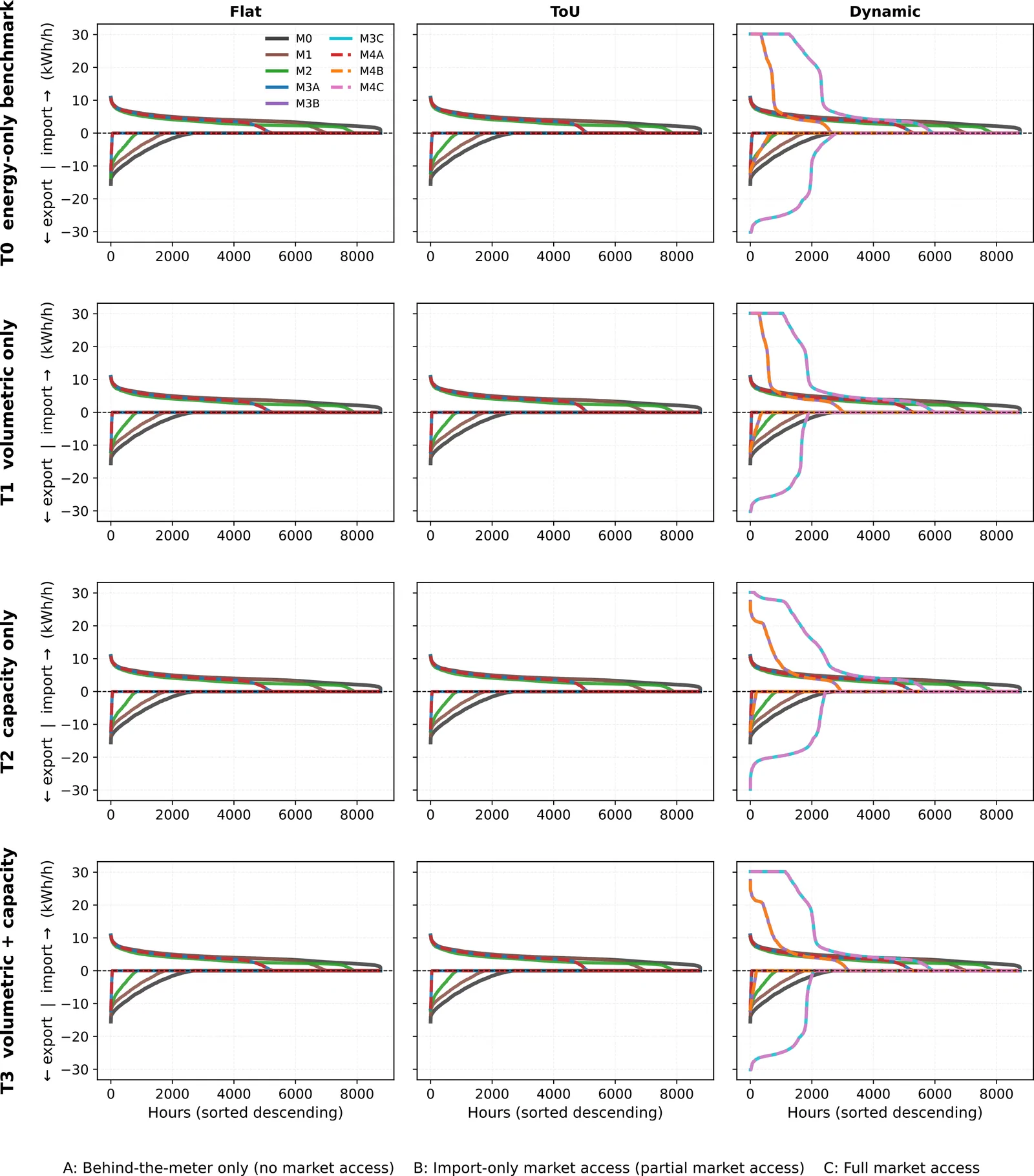
Market-integrated storage amplifies grid interaction Load-duration curves of community grid exchange across institutional configurations, the energy-only benchmark and network tariff archetypes (rows), and retail price regimes (columns). Positive values denote grid imports and negative values denote exports. Configuration and tariff definitions follow Figure 3. Settings B and C are evaluated under dynamic pricing only.

Without external market access, local exchange and storage mainly reduce surplus PV exports by increasing local electricity use. Under dynamic pricing, market access changes this operating pattern. Grid charging increases peak imports, while full market access also allows storage to discharge into the external market, increasing peak exports. Dynamic price arbitrage therefore creates additional community value but also increases peak power exchange with the grid, consistent with previous findings on the network implications of local energy trading and flexible demand.^75^^,^^77^

The 4 h storage duration is used in the main analysis. The network tariff archetypes produce similar grid exchange patterns (Figure 9). In particular, volumetric network charges have little effect on peak power, while capacity-based charges reduce some peaks where the contracted storage connection becomes binding, consistent with the findings of Nour et al.^78^ The effect of connection-capacity charging becomes more pronounced when storage power increases. To examine this relationship, Figure 10 varies storage duration from 2 to 10 h at fixed energy capacity. Shorter durations correspond to higher installed power ratings and greater ability to respond to short-term price differences.

**Figure 10:**
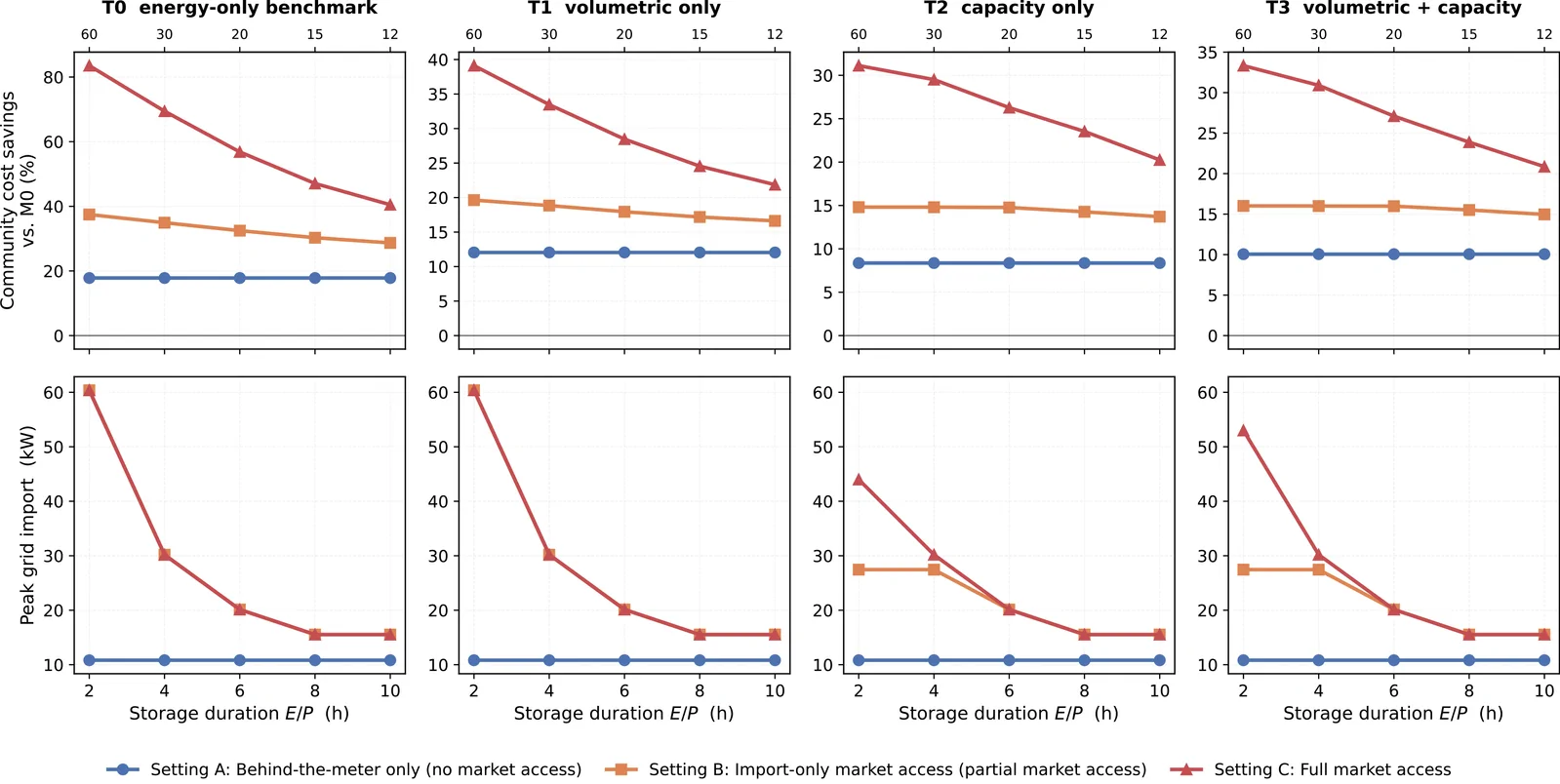
Higher storage power raises savings and peak import unless connection capacity is priced Community cost savings (top) and peak grid import (bottom) as a function of storage duration at fixed energy capacity under dynamic pricing, for community-owned shared storage. Columns show the energy-only benchmark (T0) and the three network tariff archetypes (T1–T3). The upper axis indicates the corresponding storage power rating (kW). Configuration and tariff definitions follow Figure 3.

Higher storage power increases both community savings and peak grid import when connection capacity is not priced. Under the energy-only benchmark (T0), reducing storage duration from 10 to 2 h increases community savings under full market access by 43.0 percentage points, while peak import rises from approximately 15.5 to 60.4 kW. Volumetric network tariffs reduce the economic benefit of additional power but provide little direct incentive to limit the peak.

Capacity-based tariffs change this relationship by charging for the connection capacity that storage requires. The community can reduce this cost by contracting a smaller connection, which then limits the usable storage power. At a 2 h duration, the T2 limits peak import to approximately 44 kW and the T3 to approximately 53 kW, compared with 60 kW under T0 and T1. Under T2, reducing the duration from 10 to 2 h raises community savings under full market access by only 10.9 percentage points, compared with 43.0 percentage points under T0.

Across the storage durations and network tariff archetypes examined, full market access remains the highest saving under community-owned configuration. Storage sizing therefore changes the magnitude of community savings and grid exchange without overturning the relative value of market access settings.

Overall, market access creates additional economic value but can increase peak power exchange with the grid. Volumetric network charges primarily affect the cost of energy exchanged, whereas capacity-based charges also create an incentive to limit peak power. Network tariff design therefore determines how strongly the community’s economic decisions reflect its use of grid connection capacity.

## Discussion

In the community examined, performance is mainly shaped by institutional design. By systematically separating physical system operation from economic settlement, our framework reveals how ownership structures, market access, pricing mechanisms, and network tariffs jointly determine not only how much value is created, but also how it is distributed and how it interacts with the broader power system. These findings shift the perspective on energy communities from a technical optimization problem to an institutional design problem, with direct implications for policy and market design.

### Institutional design as a system-level lever

Ownership and market access jointly shape the value of community storage. Market access determines which value streams are available, while ownership determines who captures the resulting benefits and bears the associated costs. Broader market participation can create substantial economic value, but this does not necessarily translate into greater community savings when storage is externally owned. Third-party ownership can transfer both market revenues and costs outside the community boundary.

Network tariff design further determines how storage value translates into community savings. Volumetric charges favor individual behind-the-meter batteries, whereas shared storage retains a slight advantage under contracted-capacity charging. Network tariffs also influence the cost of grid exchange and the value of additional storage power, without overturning the main ownership and market access patterns observed in this study. These interactions highlight the need to consider ownership, market access, and network tariffs jointly rather than as independent design choices.

### Distributional outcomes are shaped by institutional design, not only by the local sharing price

The local sharing price can redistribute benefits from internal energy exchange, but it cannot independently reallocate savings from individual behind-the-meter storage or market revenues captured by an external operator. These differences arise from asset ownership and the pathways through which value is created and allocated. Individual storage therefore consistently favors PV households, whereas shared storage can distribute benefits more broadly. However, the extent of this difference also depends on network tariff design. The statutory network charge reduction allows non-PV households to benefit from eligible local electricity supplied by other members, substantially reducing the group-level differences observed in the energy-only benchmark.

The local sharing price is thus one instrument for shaping distributional outcomes within a broader institutional arrangement, rather than a means of correcting every difference in value capture. The CV-minimizing sharing price is an analytical benchmark for the evenness of relative household savings, not a normative definition of fairness or a recommended tariff.

We adopt proportional allocation of locally shared electricity as a common, meter-based settlement assumption, allowing us to compare institutional configurations without also changing the allocation rule. Alternative approaches, such as the Shapley value and nucleolus, address a different question: how to divide the gains from cooperation according to the value of different member coalitions. Applying them would require specifying which storage assets, market access rights, and contractual obligations each hypothetical coalition retains, and estimating its value under those conditions. The resulting allocations would therefore depend on additional counterfactual assumptions rather than measured energy flows alone, and could not be verified by a member from its own metered data. Auction-based approaches would require bids or preferences that are not available in the case study data and could also affect dispatch, so that the allocation rule and physical operation could no longer be separated. A systematic comparison of these approaches would constitute a separate allocation-design analysis. Our household-level findings, including the CV and the PV-non-PV savings gap, are consequently conditional on the adopted allocation approach. Alternative ex-post allocations could change these outcomes without changing aggregate community savings, provided dispatch and payments to external actors remain fixed.

### Trade-offs between community economic efficiency and system-level impacts

Market access can substantially increase community savings by allowing storage to respond to external price signals, but the resulting arbitrage also increases peak imports and exports at the grid interface. The configurations that create the greatest economic value for the community can therefore require greater exchange capacity with the upstream grid. This reveals a trade-off between community-level economic benefits and the wider system implications of storage operation, as reflected in the grid-exchange patterns examined here.

Network tariff design influences how this trade-off affects community decisions. Volumetric charges increase the cost of electricity withdrawal but provide little direct incentive to limit peak power. Capacity-based charges, by contrast, make higher connection capacity more costly and can therefore limit the power used for market arbitrage. Our storage-sizing analysis confirms that the additional value of higher storage power decreases when connection capacity is priced. Coordinating market access with network tariff design can thus allow communities to capture flexibility value while accounting for the grid capacity required to support it.

### Methodological contribution

Beyond the empirical findings, this work contributes a transparent framework for analyzing how institutional arrangements shape value creation, capture, and distribution in energy communities. A key methodological contribution is the ex-post energy-flow decomposition, which traces how economic value is associated with different physical flows and how it is allocated among households, the community, storage operators, and the external grid. This makes it possible to distinguish changes in total value creation from changes in who captures that value, providing a direct link between value creation, value capture, and distribution. The same accounting framework also allows regulatory rules to be represented explicitly, such as the statutory restrictions determining which local energy flows qualify for reduced network charges. It therefore provides a consistent basis for comparing ownership structures, market access arrangements, and network-tariff designs within a common modeling framework.

The framework is designed to be transferable across energy community settings, but the quantitative results of the present study are derived from a single case study and depend on its demand and generation profiles, community composition, climatic conditions, electricity prices, and regulatory environment. What is more transferable are the mechanisms identified by the framework, for example, the distinction between value creation and value capture, the interaction between market access and ownership, and the way network tariffs translate grid use into community costs. Their quantitative importance, and potentially their relative strength, may differ across jurisdictions and community settings. Applying the framework to other energy communities therefore primarily requires context-specific adaptation of input data, regulatory rules, and technology assumptions, while the overall analytical structure remains unchanged; the transferability of the quantitative findings remains to be established in communities with different climatic, regulatory, and socio-economic conditions.

Our framework focuses on institutional dimensions that directly determine storage ownership, access to value streams, electricity prices, and the allocation of modeled costs and revenues. This focus captures economically relevant differences between arrangements observed in European energy communities, without attempting to represent their full governance structures. For example, Ecopower in Belgium illustrates a member-governed energy cooperative, while the Magliano Alpi community in Italy illustrates a municipality-initiated approach.^79^^,^^80^ Studies of European energy communities also identify third-party-sponsored and energy-service-company arrangements.^81^^,^^82^ Within these broader governance models, collective ownership of shared storage is represented by M3, whereas storage owned by an external service provider is represented by M4.

These correspondences are limited to the ownership and economic responsibilities of the storage asset; they do not imply that the cited communities operate the specific storage configurations modeled here. Household heterogeneity is represented through the measured electricity demand and PV generation profiles of each unit, and distributional outcomes are examined for both PV and non-PV households. Income and tenure status introduce further dimensions of heterogeneity, but their effects extend beyond the allocation of electricity costs. They may influence who can invest in shared assets, who chooses to participate, how costs and benefits are negotiated, how decisions are made, the trust among members, and whether a given saving is meaningful relative to a household’s financial circumstances. Assessing these effects would require modeling participation and investment decisions, governance processes, and household welfare. Our analysis instead holds community membership and the institutional arrangements under comparison as given, allowing us to examine how ownership, market access, and pricing shape value creation, capture, and distribution within the same community. The resulting savings and distributional indicators are therefore conditional economic outcomes, not measures of social equity or predictions of who will join a community and which arrangements its members will adopt.

### Institutional adaptability and path dependence

The framework compares institutional configurations as alternative states, but real energy communities move through these states over time. Ownership and contractual arrangements are often chosen under current market and regulatory conditions and may remain in place as those conditions evolve. This creates path dependence: an arrangement that is attractive when the asset is first deployed may become less favorable as new value streams, market access opportunities, or regulatory rules emerge.

Ownership is particularly important in this respect because it determines both who bears the costs associated with the asset and who captures new value when additional opportunities become available. Third-party ownership may reduce the community’s initial capital burden and transfer some operating or connection costs outside the community boundary, but it also transfers part of the future upside. Community ownership preserves that upside within the community but requires greater financial and organizational commitment from the outset. The preferred ownership model can therefore change as the economic and regulatory environment evolves, even when the underlying physical asset remains unchanged.

Network tariffs constitute another source of regulatory change over the lifetime of a storage asset, but their role in path dependence is different. In the present case, changing the structure of network charges alters the economic value of the configurations and the incentives associated with grid use, but does not overturn the broader ownership and market access patterns. Market access reform can therefore change not only how much an existing ownership arrangement is worth but also which ownership model is more advantageous, whereas network tariff reform primarily changes the magnitude of that advantage.

This suggests that institutional design should be evaluated not only in terms of current economic performance but also in terms of adaptability. Long-lived ownership and service contracts can lock communities into allocation structures that become less favorable as market access, network regulation, or other market conditions evolve. Contractual provisions such as periodic review, renegotiation clauses, or buy-out options can reduce this exposure by preserving the ability to adapt. The relevant design question is therefore not only which arrangement performs best today but also how well it can accommodate changes in the institutional environment over the lifetime of the asset.

### Implications for regulatory design

The results suggest that market access and network pricing should be designed jointly. Expanding market access allows community storage to access additional value streams, but it can also increase peak imports and exports when storage responds to external price signals. Restricting market participation would reduce these interactions but would also remove potentially valuable uses of flexibility. Network-aware tariffs provide a more targeted alternative by allowing market access while reflecting the cost of grid use in the community’s economic decision. In particular, capacity-based charges create a direct incentive to limit peak power, whereas volumetric charges primarily price the amount of energy exchanged. Regulatory design should therefore account not only for how much electricity communities exchange with the grid, but also for the connection capacity required to support that exchange.

Ownership regulation should likewise be considered together with market access rather than in isolation. There is no universally preferred ownership model: third-party ownership can reduce the financial and network-cost exposure borne directly by the community, while community ownership allows a larger share of future storage value to remain within the community. Which arrangement is more advantageous depends on the value streams available to the asset and may change as market access evolves. Regulatory frameworks should therefore preserve flexibility in ownership models and avoid assuming that one structure is optimal across all market conditions. Under third-party ownership, any positive fixed participation fee transfers value from members to the operator one-for-one, so the community savings reported for model 4 are an upper bound. The incidence of such a fee depends on how it is allocated: an equal per-member fee is regressive with respect to consumption, because flow-based benefits scale with electricity use while the fee does not. In the case study, annual consumption ranges from 2.26 to 15.86 MWh (Table 1), so an equal fee would absorb roughly seven times as large a share of the smallest member’s bill as of the largest. Third-party contracts should therefore recover costs mainly through flow-based charges or differentiate the fixed component across members.

Local sharing incentives also require careful definition of which energy flows qualify for support. Reductions in network charges can increase the value of local coordination, but the incentive should remain linked to electricity that is supplied from within the community. Electricity imported from the grid, stored, and subsequently resold internally should therefore be treated differently from locally generated electricity; otherwise, an incentive intended to reward local renewable energy use may also subsidize external market arbitrage. More broadly, our results indicate that network tariff reform primarily changes the magnitude of community savings and the incentives associated with grid use, while the dominant effects of ownership and market access remain largely unchanged.

Finally, institutional design cannot substitute for project economics. The framework identifies how different arrangements affect the annual value created, captured, and distributed within the community, and this value can be translated into an indicative level of investment cost that the project could support. This should be interpreted as economic headroom rather than a complete assessment of financial viability. Actual deployment also depends on capital costs, degradation, operation and maintenance, financing conditions, and additional revenue streams outside the present model. Institutional design therefore determines how available value is organized and allocated, but not whether storage is economically viable under all real-world conditions.

### Limitations of the study

Several limitations should be considered when interpreting the results.

First, the analysis represents network costs through an energy-only benchmark and three network tariff archetypes. The network tariffs are calibrated *ex ante* to recover the same network revenue in the reference case, but realized revenues may differ when storage operation changes. Actual network tariffs and the eligibility rules for local network-charge reductions also vary across regulatory contexts. These differences could influence both economic outcomes and distributional effects. Moreover, grid impacts are assessed through power exchange at the community interface, without explicitly modeling network congestion or voltage conditions.

Second, the case study is based on real-world load and PV generation data, which enhances realism but represents a static system configuration. Future changes in technology adoption, such as increased PV penetration or electrification, as well as changes in community composition, may alter the relative performance of different institutional designs. The analysis compares alternative institutional states and does not simulate household entry and exit, regulatory transitions, or the costs of changing ownership arrangements.

Third, storage energy capacity is set equal to one day of average community demand and held constant across configurations to isolate the effects of institutional design. This demand-based sizing rule provides a consistent basis for comparison but may not reflect the capacity best suited to each institutional arrangement. Different energy capacities could affect storage utilization, cost savings, and grid exchange, potentially changing the magnitude and relative performance of the modeled configurations. The annual optimization also assumes perfect foresight. A rolling-horizon test with 24-h commitments and a 48-h look-ahead increases annual community cost by at most 1.65% (STAR Methods; Table 6); forecast errors in demand and PV generation are not modeled in this study.

**Table 6:** Increase in annual community cost under rolling-horizon dispatch relative to the annual perfect-foresight solution, dynamic pricing

Archetype	Setting A		Setting B		Setting C	
	24 h (%)	48 h (%)	24 h (%)	48 h (%)	24 h (%)	48 h (%)
T0 energy-only benchmark	5.13	1.65	14.82	1.30	2.24	0.01
T1 volumetric only	3.14	0.88	6.93	0.90	1.55	0.81
T2 capacity only	3.37	0.93	5.88	0.82	1.33	0.63
T3 volumetric + capacity	3.29	0.90	6.01	0.81	1.43	0.77

Community cost includes energy purchases net of export revenue, household network charges net of the statutory reduction, and the storage connection charge net of its reimbursement.

Finally, the baseline third-party storage model assumes a zero fixed participation fee and an exogenous per-kWh service fee to isolate value capture through operational flows. Additional contractual payments would affect the distribution of value between operators and community members. As the operator’s full cost structure is not represented, the estimated economic headroom should not be interpreted as a complete assessment of project profitability.

## Resource availability

### Lead contact

Requests for further information and resources should be directed to and will be fulfilled by the lead contact, Na Li (na.li@empa.ch).

### Materials availability

This study did not generate new materials.

### Data and code availability

• The data used in this study consist of real-world electricity demand and PV generation profiles from the vertical energy district at NEST building at Empa (Switzerland). The processed datasets used for the analysis have been deposited on Zenodo (https://doi.org/10.5281/zenodo.21132071) and are publicly available as of the date of publication. This paper also analyzes existing, publicly available data. The day-ahead electricity prices for the Swiss bidding zone were obtained from the ENTSO-E Transparency Platform (https://transparency.entsoe.eu/); the retail energy and Swiss network tariff sheets and the feed-in remuneration rates were obtained from Glattwerk AG (https://www.glattwerk.ch/ and https://www.glattwerk.ch/downloads); the capacity-based network tariff for small consumers were obtained from Stedin Netbeheer B.V. (https://www.stedin.net/); and the Swiss Electricity Supply Ordinance (StromVV, SR 734.71) was obtained from the Swiss Federal Council (https://www.fedlex.admin.ch/eli/oc/2025/139/de). These sources are listed in the key resources table.
• All original code used to generate the results presented in this study is publicly available in the same Zenodo repository (https://doi.org/10.5281/zenodo.21132071) and is accessible as of the date of publication. The same code is maintained at https://github.com/nali-delft/institutional-design-community-energy-storage; the Zenodo deposit is the archived version of record. Both links are listed in the key resources table. The code runs on Python 3.13 (https://www.python.org/) and the optimization models are solved with Gurobi Optimizer 13.0.1 (https://www.gurobi.com/), which requires a separate license.
• Any additional information required to reanalyze the data reported in this paper is available from the lead contact upon request.

## Acknowledgments

This research is conducted within HORIZON EUROPE SOCIAREM project and has received funding from the European Union’s Horizon Europe research and innovation programme under grant agreement for project no. N° 101235482 and the Swiss Secretariat for Education, Research, and Innovation (SERI) under contract no. N°25.00325.

## Author contributions

Conceptualization, N.L.; methodology, N.L.; investigation, N.L.; writing – original draft, N.L.; writing – review and editing, N.L. and B.K.; funding acquisition, B.K.; resources, N.L. and B.K.

## Declaration of interests

The authors declare no competing interests.

## Declaration of generative AI and AI-assisted technologies in the manuscript preparation process

During the preparation of this work, the authors used Claude and ChatGPT to assist with drafting and language refinement. The authors reviewed and edited the output as needed and take full responsibility for the content of the published article.

## STAR★Methods

### Key resources table

**Table:** 

REAGENT or RESOURCE	SOURCE	IDENTIFIER
**Deposited data**		

Measured hourly electricity demand and PV generation profiles of seven units of the NEST building, calendar year 2025	This paper	Zenodo: https://doi.org/10.5281/zenodo.21132071
Model results for the 76 institutional scenarios	This paper	Zenodo: https://doi.org/10.5281/zenodo.21132071
Day-ahead electricity prices, Swiss bidding zone, 2025	ENTSO-E Transparency Platform^84^	https://transparency.entsoe.eu/
Retail energy and network tariff sheets, valid from 1 January 2026	Glattwerk AG^83^^,^^85^	https://www.glattwerk.ch/
Feed-in remuneration rates, valid from 1 July 2025	Glattwerk AG^86^	https://www.glattwerk.ch/downloads
Small-consumer electricity network tariffs 2026	Stedin Netbeheer B.V.^87^	https://www.stedin.net/
Swiss Electricity Supply Ordinance (StromVV), SR 734.71, Art. 19g and 19h	Swiss Federal Council^76^	https://www.fedlex.admin.ch/eli/oc/2025/139/de

**Software and algorithms**		

Original code for the institutional design framework (models, settlement, figures)	This paper	Zenodo: https://doi.org/10.5281/zenodo.21132071; GitHub: https://github.com/nali-delft/institutional-design-community-energy-storage
Python 3.13	Python Software Foundation	https://www.python.org/
Gurobi Optimizer 13.0.1	Gurobi Optimization, LLC	https://www.gurobi.com/

### Method details

This study develops an integrated modeling framework to systematically evaluate how institutional design shapes the performance of community energy systems. Rather than focusing on a single configuration, we treat institutional design itself, specifically ownership structure, market integration, retail price regime, and network tariff design, as a set of key decision variables.

The framework integrates three layers within a consistent structure: physical system operation, economic settlement, and distributional outcomes. Physical dispatch is determined by external price signals and system constraints, while internal settlement mechanisms are applied ex post to allocate costs and benefits across community members. This separation allows efficiency, distributional outcomes, and value capture to be analyzed independently under different institutional arrangements.

The method details are organized as follows. We first describe the case study and the measured data. We then define the institutional design space and corresponding model configurations, including the network tariff archetypes, the electricity price regimes, and the internal settlement mechanism and the evaluation metrics. We then give the complete mathematical formulation of the five models, followed by the price, tariff, and regulatory parameters used in the case study and the robustness analyses.

#### Case study and data

The case study, NEST, is a modular research and innovation vertical energy district, located at the Empa campus in Dübendorf, Switzerland.^88^ NEST comprises ten building units. We include the seven units for which complete hourly electricity demand and PV generation profiles are available for the full 2025 calendar year; the remaining three units are excluded because their records are incomplete. The seven retained units define the empirical community analyzed here. Their annual electricity consumption and PV generation profiles are summarized in Table 1 and Figure 1.

The selected units provide the heterogeneity needed to examine institutional design. Annual electricity consumption ranges from 2.26 to 15.86 MWh, and three of the seven units have PV systems. Aggregate PV generation is 22.24 MWh, equal to 50% of aggregate consumption, but generation and demand are not matched in time: generation exceeds consumption in summer and is substantially lower in winter. The units also differ in the shape of their demand: the ratio of mean weekday to mean weekend consumption ranges from 0.95 to 1.82, and the hour of the average daily peak from 08:00 to 18:00. Demand peaks are accordingly non-coincident, the sum of the individual peaks being 23.6 kW against a community coincident peak of 12.1 kW. This temporal and spatial diversity creates the pooling opportunity through which shared storage can serve one unit’s demand using another unit’s surplus.

Consumption and PV generation are measured hourly over calendar year 2025. The analysis treats the seven retained profiles as fixed inputs and compares how the cost of serving them is created, captured and allocated under different institutional arrangements.

#### Institutional design space

The institutional design of energy communities is characterized along four dimensions: ownership structure, the degree of market integration permitted to shared assets, the retail price regime, and network tariff design. Ownership determines who controls the storage asset and who captures its economic value, ranging from no shared asset, through individual ownership, to collective community ownership, and finally to third-party ownership. The degree of market integration determines whether shared assets are restricted to internal community balancing or are allowed to interact with external electricity markets. The retail price regime determines the temporal price signal to which storage operation responds. Network tariff design determines how the community’s use of the distribution network is charged and whether the tariff provides volumetric or capacity-based incentives. The resulting design space is illustrated in Figure 2, which integrates physical system operation, institutional configurations, network tariff design and economic settlement within a single representation.

This paper systematically spans this design space. Along the ownership dimension, we consider five model configurations: a reference case without an energy community (Model 0), an energy community with local sharing but no storage (Model 1), an energy community with individual-owned energy storage (Model 2), an energy community with community-owned energy storage (Model 3), and an energy community with a third-party-owned energy storage (Model 4). For configurations with community-level storage (Models 3 and 4), we further distinguish three institutional settings governing market access: a behind-the-meter coordination regime in which storage is restricted to internal balancing (Setting A), a market-enabled procurement regime permitting grid charging but not grid export (Setting B), and a fully market-integrated regime with unrestricted grid interaction (Setting C). The retail price regime and the network tariff archetype are varied independently of both, through the three price regimes, the energy-only benchmark, and the three network tariff archetypes defined below. The five configurations without market access are evaluated under all three price regimes and the four market access configurations (M3B, M3C, M4B, M4C) under dynamic pricing only, giving (5 × 3 + 4 × 1) × 4 = 76 scenarios (Figure 3).

Together, these configurations constitute a structured scan of the institutional design space, as illustrated in Table 2.

These configurations are not merely theoretical constructs but reflect institutional arrangements that are actively emerging or under regulatory discussion across European energy markets. Setting A corresponds to current behind-the-meter regulatory frameworks, Setting B reflects emerging proposals for limited aggregator access, and Setting C represents fully market-integrated operation, as studied in virtual power plant and storage-as-a-service models. This formulation enables a systematic comparison of how regulatory choices affect value creation, distribution, and capture.

#### Energy price regimes

The retail price regime is the third dimension of the design space; we consider three regimes. Under a flat tariff, households face a constant retail price and feed-in tariff throughout the day, representing the simplest and still most common retail structure in many European retail markets. Under a time-of-use (ToU) tariff, prices vary between predefined peak and off-peak periods, reflecting the broad temporal structure of system costs. Under a dynamic tariff, retail prices follow hourly wholesale market prices, exposing households and storage operators to the full short-run price signals. These three regimes span a range from minimal to strong price-based incentives, and allow us to examine how the value of different institutional configurations interacts with the richness of the price signals.

#### Network tariff regimes

Network tariff design is treated as a separate institutional dimension because it determines how grid use is charged and how storage operation responds to network costs. We consider an energy-only benchmark and three network tariff archetypes. T0 is the energy-only benchmark, which excludes network charges to isolate the energy component of community costs. T1 represents the tariff at the case study location, with volumetric charges on grid withdrawals. T2 applies charges to contracted connection capacity, which also limits storage charging and discharging power. T3 combines volumetric and contracted-capacity charges. T1–T3 are calibrated ex ante to recover the same network revenue from the reference community (M0), allowing the effects of tariff structure to be compared at a common baseline revenue level. Realized network revenues may nevertheless differ as community operation changes. Tariff parameters, connection-capacity options, regulatory assumptions, and the calibration procedure are given under network tariff archetypes in STAR Methods (Tables 4 and 5).

#### Internal settlement design

##### Local sharing price and distributional outcome criterion

Internal energy transactions within the community are settled at a local sharing price $\pi_{t}^{\text{loc}}$, which is set to satisfy: $\pi_{t}^{\text{exp}}\leq \pi_{t}^{\text{loc}}\leq \pi_{t}^{\text{imp}}$. This condition ensures individual rationality: PV households receive a higher return than exporting to the grid, while consuming households pay less than purchasing from the grid, making participation in local sharing beneficial for all members, regardless of their asset ownership. In the numerical analysis we set *π*^exp^ = 0.06 CHF/kWh, the statutory minimum feed-in remuneration for PV installations below 30 kW, which binds for every system in the case study,^86^ and take the upper bound from the flat energy price of 0.1229 CHF/kWh derived under electricity price regimes in STAR Methods (Table 3). All monetary values are in Swiss francs excluding VAT.

A key feature of the framework is the separation between physical system operation and internal economic settlement. Specifically, the local sharing price does not affect operational dispatch decisions in any model. Physical operation is determined solely by external import and export prices. Local sharing price affects only the ex-post cost allocation among households. This separation of physical dispatch from internal settlement ensures that the choice of sharing price has no effect on total community electricity expenditure, it governs only how that expenditure is distributed across members.

Within the feasible range, we evaluate sharing prices at increments of 0.01 CHF/kWh, yielding *π*^loc^ ∈ {0.06, 0.07, …, 0.12} CHF/kWh. The distributional outcome-optimal sharing price is selected as the value that minimizes the CV of relative electricity cost savings across all community members:

(Equation 1) $$\pi^{\text{loc},*}=\arg\min_{\pi^{\text{loc}}}\;CV\;\left({\left\{\frac{C_{i}^{\text{M0}}-C_{i}^{\text{Mx}}}{C_{i}^{\text{M0}}}\right\}}_{i\in N}\right),\;CV\left(\cdot \right)=\frac{\sigma \left(\cdot \right)}{\mu \left(\cdot \right)}.$$

This criterion selects the price at which the percentage reduction in electricity costs is most uniformly distributed across households, ensuring that community benefits are accessible to members regardless of PV ownership status and without affecting system efficiency.

##### Internal settlement

In the reference case (Model 0), no internal settlement takes place. In Models 1 and 2, settlement is straightforward: locally shared PV energy is allocated proportionally based on residual demand and surplus, and each household’s cost is determined directly by the combination of local and grid transactions at their respective prices. No energy flow decomposition is required, as storage either does not exist (Model 1) or operates entirely behind the individual household meter (Model 2). We adopt proportional allocation for three reasons. It is meter-based and transparent: each household’s share of locally exchanged electricity follows directly from measured flows and can be reproduced by any member from its own meter data. It is applied ex post and does not influence dispatch, so it leaves community-level efficiency unchanged and can be held fixed across all configurations to isolate the effects of ownership, market access, pricing, and network tariffs. It also coincides with the attribution key that Swiss distribution system operators apply when determining participants’ network charges and basic-supply volumes (Art. 19g(4)–(5) StromVV^76^), so that internal settlement is consistent with external billing; the ordinance itself does not regulate how a community shares costs and benefits among its members.

In Models 3 and 4, community-level storage is present, and the proportional method is extended through an explicit ex-post energy flow decomposition. After physical dispatch, realized energy flows are attributed using a priority-based accounting rule: direct local PV sharing is settled first, followed by PV charging of the battery, grid charging of the battery, battery discharge to local load, and grid export flows. This decomposition serves different roles depending on ownership. In Model 3 (community-owned storage), it enables the separation of storage-related revenues and costs into a dedicated CES settlement account, which is then redistributed to households according to ownership shares. In Model 4 (third-party storage), the same decomposition ensures that storage service fees and revenues apply only to flows involving the storage system, enabling transparent value capture by the third-party operator. In both models, dispatch minimizes community-level cost; the third-party operator is assumed to adopt the same operating objective, so that ownership affects only who captures the resulting revenues and bears the costs. Importantly, direct local sharing bypasses the storage system and does not contribute to operator revenue.

The complete mathematical formulation of all five models, including the energy flow decomposition rules and household cost calculation, is given in method details below.

#### Evaluation metrics

We evaluate the performance of institutional configurations along three complementary dimensions: community economic performance, the distribution of household savings, and system-level impact. These metrics correspond to the three core questions addressed in the results section: how much value is created, how it is distributed, and how it affects the upstream grid. These dimensions reflect the trade-offs inherent in institutional design, linking value creation, distribution, and system integration.

##### Economic performance

Community cost is defined as the aggregate annual net electricity expenditure borne by community members after all energy transactions and settlements. It includes payments for electricity purchased from external suppliers and markets, applicable network charges after statutory reductions, and payments to external storage operators. Revenues from electricity exports and market transactions that accrue to the community are deducted. Under community-owned storage (M3), the storage connection charges and operating revenues and costs are included in community cost. Under third-party ownership (M4), payments to the operator are included, but the operator’s own revenues and costs are reported separately and are not included in community cost.

For each configuration *m*, community cost savings are calculated relative to the reference case (M0) under the same retail price regime and network tariff:

(Equation 2) $$S_{m}^{\text{com}}=\frac{C_{0}^{\text{com}}-C_{m}^{\text{com}}}{C_{0}^{\text{com}}}\times 100\%,$$

where $C_{m}^{\text{com}}$ denotes annual community cost under configuration *m*. Storage investment, degradation, and replacement costs are not included in this operating-cost metric.

##### Distributional outcomes

The distribution of economic benefits across community members is evaluated using two complementary measures. First, the CV of relative household cost savings, Equation 1, measures how evenly savings are distributed across households. A lower CV indicates a more even distribution of relative savings. Because the CV captures overall variation but does not indicate which groups benefit more, it is complemented by a comparison of the average relative savings of PV and non-PV households. The difference between these group averages indicates whether the benefits of a given configuration are concentrated more strongly among households with or without PV.

##### System-level impact

System-level impact is evaluated through the community’s net electricity exchange with the grid, characterized using load duration curves. Key indicators include peak import and export levels, as well as the distribution of grid exchange over time. This metric captures how institutional configurations influence grid stress and variability.

##### Third-party financial position

For configurations with third-party-owned storage (Model 4), we additionally report the operator’s financial position to assess how institutional design affects value capture by external actors. It comprises storage service revenue, the market arbitrage margin, and the network charge on the storage connection. It is an operating position: it excludes the fixed participation fee, which is set to zero in the baseline analysis, and the operator’s investment and operating costs.

#### Model notation and common definitions

##### Sets and indices

Let N denote the set of households in the community, indexed by *i*, and T the set of time periods, indexed by *t*. The subset $N^{\text{B}}\subseteq N$ denotes households equipped with individual battery storage. In the present study, battery ownership is restricted to PV-owning households, so households in $N\setminus N^{\text{B}}$ neither own PV nor storage.

##### Exogenous parameters

• *d*_*i*,*t*_: electricity demand of household *i* at time *t*
• *p*_*i*,*t*_: PV generation of household *i* at time *t*
• $\pi_{t}^{\text{imp}}$: retail import price at time *t*
• $\pi_{t}^{\text{exp}}$: feed-in tariff (export price) at time *t*
• $\pi_{t}^{\text{loc}}$: internal local sharing price, satisfying $\pi_{t}^{\text{exp}}<\pi_{t}^{\text{loc}}<\pi_{t}^{\text{imp}}$ for all *t*
• *M*^grid^: big-M constant for grid exchange constraints

##### Battery parameters

• *η*^ch^, *η*^dis^: charging and discharging efficiency
• *E* (or *E*_*i*_): usable energy capacity
• *P*^ch^ (or $P_{i}^{\text{ch}}$): maximum charging power
• *P*^dis^ (or $P_{i}^{\text{dis}}$): maximum discharging power

##### Quantities common to all models

Self-consumption, residual surplus, and residual demand are defined identically across Models 0–4:

(Equation 3) $$x_{i,t}^{\text{self}}=\min\left(d_{i,t},\;p_{i,t}\right),$$

(Equation 4) $${\tilde{p}}_{i,t}^{\text{sur}}=p_{i,t}-x_{i,t}^{\text{self}},$$

(Equation 5) $${\tilde{d}}_{i,t}^{\text{res}}=d_{i,t}-x_{i,t}^{\text{self}}.$$

Community-level aggregates (used in Models 1, 3, and 4) are

(Equation 6) $$P_{t}^{\text{sur}}=\underset{i\in N}{\sum }{\tilde{p}}_{i,t}^{\text{sur}},\;D_{t}^{\text{res}}=\underset{i\in N}{\sum }{\tilde{d}}_{i,t}^{\text{res}}.$$

##### Proportional allocation rule

In all models with local sharing, locally exchanged energy is allocated proportionally:

(Equation 7) $$x_{i,t}^{\text{buy}}=\frac{{\tilde{d}}_{i,t}^{\text{res}}}{D_{t}^{\text{res}}}\;x_{t}^{\text{loc}},\;D_{t}^{\text{res}}>0,$$

(Equation 8) $$x_{i,t}^{\text{sell}}=\frac{{\tilde{p}}_{i,t}^{\text{sur}}}{P_{t}^{\text{sur}}}\;x_{t}^{\text{loc}},\;P_{t}^{\text{sur}}>0.$$

Both quantities equal zero when the respective denominator is zero.

##### Battery dynamics (shared structure)

Whenever a battery appears, individual (Model 2) or community (Models 3–4), the state of charge (SoC) evolves as

(Equation 9) $$s_{t}=s_{t-1}+\eta^{\text{ch}}x_{t}^{\text{ch}}-\frac{1}{\eta^{\text{dis}}}x_{t}^{\text{dis}},\;\forall t,$$

subject to

(Equation 10) $$0\leq s_{t}\leq E,$$

(Equation 11) $$0\leq x_{t}^{\text{ch}}\leq P^{\text{ch}}y_{t}^{\text{ch}},$$

(Equation 12) $$0\leq x_{t}^{\text{dis}}\leq P^{\text{dis}}y_{t}^{\text{dis}},$$

(Equation 13) $$y_{t}^{\text{ch}}\;+y_{t}^{\text{dis}}\leq 1,\;y_{t}^{\text{ch}},\;y_{t}^{\text{dis}}\in \left\{0,1\right\}.$$

No simultaneous grid import and export is enforced by

(Equation 14) $$0\leq x_{t}^{\text{imp}}\leq M^{\text{grid}}\;y_{t}^{\text{grid}},$$

(Equation 15) $$0\leq x_{t}^{\text{exp}}\leq M^{\text{grid}}\;\left(1-y_{t}^{\text{grid}}\right),\;y_{t}^{\text{grid}}\in \left\{0,1\right\}.$$

Battery parameterization and initial conditions. The energy storage system is sized proportionally to aggregate community load, with an energy capacity of $E={\bar{d}}^{\text{annual}}/365$kWh and a power rating of *P* = *E*/4 kW, reflecting a 4-h storage duration consistent with residential-scale systems. For individual batteries in Model 2, sizing is based on each household’s annual load.

The initial SoC is set to *s*_0_ = 0 in all models. A cyclic terminal constraint *s*_*T*_ = *s*_0_ is imposed to ensure that the optimization horizon does not create artificial end-of-period discharge incentives and to ensure comparability of results across models and price regimes. This constraint implies that the storage system neither accumulates nor depletes energy over the full simulation horizon, which is consistent with a steady-state operational assumption.

##### Network charges

Network charges are assessed at each metering point based on electricity withdrawn from the distribution grid. For household *i*, the metered withdrawal is its residual demand ${\tilde{d}}_{i,t}^{\text{res}}$ in Equation 5, defined as net load of on-site self-consumption. In Model 2, where the battery is behind the household meter, withdrawal is instead the post-battery import $x_{i,t}^{\text{imp,}1}$. We denote metered withdrawal in either case by $x_{i,t}^{\text{draw}}$. Electricity received through local sharing or discharged from shared storage is included in the receiving household’s withdrawal and is therefore subject to network charges, with any applicable statutory reduction calculated below.

The gross annual network charge for household *i*, with contracted connection capacity ${\bar{C}}_{i}$, is

(Equation 16) $$N_{i}=\underset{\text{volumetric}}{\underset{︸}{\underset{t\in T}{\sum }c_{t}^{\text{net}}x_{i,t}^{\text{draw}}}}+\underset{\text{capacity}}{\underset{︸}{\kappa \;{\bar{C}}_{i}}}+\underset{\text{fixed}\;\text{and}\;\text{metering}}{\underset{︸}{12\left(f+m\right)}},$$

where $c_{t}^{\text{net}}$ is the volumetric network charge, *κ* is the annual charge per kilowatt of contracted capacity, and *f* and *m* are the monthly fixed and metering charges, respectively. T1 applies volumetric charging (*κ* = 0), T2 applies capacity charging $\left(c_{t}^{\text{net}}=0\right)$, and T3 combines both. All network-charge components are set to zero under the energy-only benchmark T0 (see network tariff archetypes below).

In the context of this case study located in Switzerland, eligible electricity supplied within an energy community receives a statutory reduction at rate *δ* on the network charge as stated in Article 19h of the StromVV (40% when no voltage transformation is required between generation and consumption),^76^ excluding metering:

(Equation 17) $$R_{i}^{\text{EC}}=\left(\underset{t\in T}{\sum }c_{t}^{\text{net}}x_{i,t}^{\text{draw}}+\kappa \;{\bar{C}}_{i}+12f\right)\frac{E_{i}^{\text{EC}}}{E_{i}^{\text{draw}}}\;\delta ,\;E_{i}^{\text{draw}}=\underset{t\in T}{\sum }x_{i,t}^{\text{draw}},$$

where $E_{i}^{\text{EC}}$ is the eligible electricity supplied to household *i* from within the community:

(Equation 18) $$E_{i}^{\text{EC}}=\underset{t\in T}{\sum }x_{i,t}^{\text{buy,loc}}+\varphi \underset{t\in T}{\sum }x_{i,t}^{\text{dis,load}},\;\varphi =\min\;\left(1,\;\frac{\underset{t}{\sum }x_{t}^{\text{ch,pv}}}{\underset{t}{\sum }x_{t}^{\text{dis,load}}}\right).$$

The factor *φ* caps eligible storage-mediated supply over the billing period at the amount of locally generated electricity charged into shared storage. Thus, grid-charged electricity does not create additional eligibility for the reduction. The denominator in Equation 17 is metered withdrawal rather than total household consumption, consistent with the basis on which the network charge is assessed. The household’s net network cost is

(Equation 19) $$N_{i}^{\text{net}}=N_{i}-R_{i}^{\text{EC}}.$$

For zero annual withdrawal, we set $R_{i}^{\text{EC}}=0$; for zero storage discharge to local load, we set *φ* = 0. Under T0, $N_{i}^{\text{net}}=0$.

In Models 3 and 4, shared storage has a separate grid connection and is charged independently of the participating households. Its annual network cost includes charges on electricity used for charging, a credit for electricity discharged, and charges for maintaining its grid connection:

(Equation 20) $$N^{\text{CES}}=\underset{t\in T}{\sum }c_{t}^{\text{net}}x_{t}^{\text{ch}}-\rho \underset{t\in T}{\sum }x_{t}^{\text{dis}}+\kappa \;{\bar{C}}^{\text{CES}}+12\left(f+m\right),$$

(Equation 21) $$x_{t}^{\text{ch}}\leq {\bar{C}}^{\text{CES}},\;x_{t}^{\text{dis}}\leq {\bar{C}}^{\text{CES}},\;{\bar{C}}^{\text{CES}}\in B,$$

where *ρ* is the network-charge credit per kilowatt-hour of electricity discharged from storage. ${\bar{C}}^{\text{CES}}$ is the contracted power capacity of the storage connection, which limits how much power the storage can charge or discharge at any time. The capacity is selected from the available connection sizes in B.

The discharge credit accounts for network charges associated with electricity passing through the storage connection before being supplied to households or exported. Eligible local electricity receives the statutory LEG reduction at the receiving household’s meter, not at the storage connection. Parameter values and the calculation of *ρ* are provided in network tariff archetypes and network tariff reduction for local supply below.

##### Model 0: reference case

Model 0 serves as the benchmark in which households operate fully independently, without local electricity sharing or coordinated asset operation. Households self-consume PV when available and exchange residual electricity with the grid.

Self-consumption and grid exchanges follow directly from Equations 3, 4, and 5:

(Equation 22) $$x_{i,t}^{\text{imp}}={\tilde{d}}_{i,t}^{\text{res}},$$

(Equation 23) $$x_{i,t}^{\text{exp}}={\tilde{p}}_{i,t}^{\text{sur}}.$$

Here $x_{i,t}^{\text{imp}}$ is also the withdrawal $x_{i,t}^{\text{draw}}$ entering Equation 16. The annual electricity cost of household *i* is

(Equation 24) $$C_{i}^{\text{M0}}=\underset{t\in T}{\sum }\left(\pi_{t}^{\text{imp}}\;x_{i,t}^{\text{imp}}-\pi_{t}^{\text{exp}}\;x_{i,t}^{\text{exp}}\right)+N_{i}.$$

##### Model 1: community without storage

Model 1 isolates the value of local electricity sharing alone. Households form an energy community that enables local sharing, but no storage assets are available. All households remain passive consumers/prosumers with respect to external markets.

##### Local sharing

The total locally exchanged electricity is

(Equation 25) $$x_{t}^{\text{loc}}=\min\;\left(P_{t}^{\text{sur}},\;D_{t}^{\text{res}}\right).$$

Individual allocations follow the proportional rule, Equations 7 and 8.

##### Residual grid exchange

(Equation 26) $$x_{i,t}^{\text{imp}}={\tilde{d}}_{i,t}^{\text{res}}-x_{i,t}^{\text{buy}},$$

(Equation 27) $$x_{i,t}^{\text{exp}}={\tilde{p}}_{i,t}^{\text{sur}}-x_{i,t}^{\text{sell}}.$$

##### Household electricity cost

The withdrawal is unchanged from Model 0, $x_{i,t}^{\text{draw}}={\tilde{d}}_{i,t}^{\text{res}}$: local exchange alters where the electricity comes from, not how much passes the meter. The gross network charge is therefore identical to Model 0’s and the whole network-side effect of membership is the reduction $R_{i}^{\text{LEG}}$, which here rests on direct exchange alone since there is no storage ($\sum_{t}x_{i,t}^{\text{dis,load}}=0$ in Equation 18).

(Equation 28) $$C_{i}^{\text{M}1}=\underset{t\in T}{\sum }\left(\pi_{t}^{\text{imp}}\;x_{i,t}^{\text{imp}}+\pi_{t}^{\text{loc}}\;x_{i,t}^{\text{buy}}-\pi_{t}^{\text{loc}}\;x_{i,t}^{\text{sell}}-\pi_{t}^{\text{exp}}\;x_{i,t}^{\text{exp}}\right)+N_{i}^{\text{net}}.$$

##### Model 2: Individual-owned storage

Model 2 introduces individual behind-the-meter battery storage for PV-owning households ($i\in N^{\text{B}}$). Batteries are optimized independently to minimize each household’s own electricity cost prior to community sharing. Battery energy is neither exported to the grid nor traded within the community; local sharing applies only to residual grid positions after individual battery operation.

##### Stage 1: Individual battery optimization

For each $i\in N^{\text{B}}$, the battery dynamics and constraints follow Equations 9, 10, 11, 12, and 13 with household-specific parameters $\left(E_{i},P_{i}^{\text{ch}},P_{i}^{\text{dis}}\right)$.

The energy balance prior to community sharing is

(Equation 29) $$d_{i,t}+x_{i,t}^{\text{ch}}+x_{i,t}^{\text{exp,}1}=p_{i,t}+x_{i,t}^{\text{dis}}+x_{i,t}^{\text{imp,}1},\;\forall i\in N^{\text{B}},\;\forall t.$$

The behind-the-meter constraint prevents grid-assisted charging and grid discharge:

(Equation 30) $$x_{i,t}^{\text{imp,}1}\leq M^{\text{grid}}\left(1-y_{i,t}^{\text{ch}}\right),$$

(Equation 31) $$x_{i,t}^{\text{exp,}1}\leq M^{\text{grid}}\left(1-y_{i,t}^{\text{dis}}\right).$$

No simultaneous grid import/export is enforced analogously to Equations 14 and 15.

Each battery-owning household minimizes its individual electricity expenditure:

(Equation 32) $$\min\underset{t\in T}{\sum }\left(\left(\pi_{t}^{\text{imp}}+c_{t}^{\text{net}}\right)x_{i,t}^{\text{imp,}1}-\pi_{t}^{\text{exp}}\;x_{i,t}^{\text{exp,}1}\right),\;\forall i\in N^{\text{B}},$$

For the household’s network charge, only the volumetric component enters this objective, because it is the only component the battery can change: the capacity, fixed and metering components are constants for the household’s fixed connection, and the statutory reduction is settled at community level in Stage 2. All components enter the household’s cost in Equation 36.

For households without storage $\left(i\notin N^{\text{B}}\right)$, Stage 1 reduces to $x_{i,t}^{\text{imp,}1}=d_{i,t}$ and $x_{i,t}^{\text{exp,}1}=0$.

##### Stage 2: Community sharing

After individual battery operation, residual positions available for sharing are

(Equation 33) $${\tilde{p}}_{i,t}^{\text{sur,}1}=x_{i,t}^{\text{exp,}1},\;{\tilde{d}}_{i,t}^{\text{res,}1}=x_{i,t}^{\text{imp,}1}.$$

Community-level totals, local exchange, and proportional allocation follow Equations 6, 7, and 8, replacing $\left({\tilde{p}}_{i,t}^{\text{sur}},{\tilde{d}}_{i,t}^{\text{res}}\right)$ with $\left({\tilde{p}}_{i,t}^{\text{sur,}1},{\tilde{d}}_{i,t}^{\text{res,}1}\right)$.

Final grid positions are

(Equation 34) $$x_{i,t}^{\text{imp}}={\tilde{d}}_{i,t}^{\text{res,}1}-x_{i,t}^{\text{buy}},$$

(Equation 35) $$x_{i,t}^{\text{exp}}={\tilde{p}}_{i,t}^{\text{sur,}1}-x_{i,t}^{\text{sell}}.$$

##### Household electricity cost

Model 2 is the one configuration in which the withdrawal itself is a decision. The battery sits behind the household’s meter, so what passes that meter is the post-battery import, $x_{i,t}^{\text{draw}}=x_{i,t}^{\text{imp,}1}$, and the battery can reduce it. This is why behind-the-meter storage lowers the gross network charge while shared storage does not, and why the saving is available only where the tariff has a volumetric component. The household’s connection capacity ${\bar{C}}_{i}$ is the standard household rating and is not a decision; it does bound the withdrawal, $x_{i,t}^{\text{imp,}1}\leq {\bar{C}}_{i}$ and $x_{i,t}^{\text{exp,}1}\leq {\bar{C}}_{i}$.

(Equation 36) $$C_{i}^{\text{M}2}=\underset{t\in T}{\sum }\left(\pi_{t}^{\text{imp}}\;x_{i,t}^{\text{imp}}+\pi_{t}^{\text{loc}}\;x_{i,t}^{\text{buy}}-\pi_{t}^{\text{loc}}\;x_{i,t}^{\text{sell}}-\pi_{t}^{\text{exp}}\;x_{i,t}^{\text{exp}}\right)+N_{i}^{\text{net}}.$$

##### Community energy storage: Models 3 and 4

Models 3 and 4 both feature a single CES system. The physical model is identical across both; they differ only in ownership, operational objective, and ex-post settlement.

##### CES dynamics and constraints

The CES SoC follows Equations 9, 10, 11, 12, and 13 with community-level parameters (*E*, *P*^ch^, *P*^dis^).

##### Community energy balance

(Equation 37) $$P_{t}^{\text{sur}}+x_{t}^{\text{imp}}+x_{t}^{\text{dis}}=D_{t}^{\text{res}}+x_{t}^{\text{ch}}+x_{t}^{\text{exp}},\;\forall t.$$

No simultaneous grid import/export is enforced by Equations 14 and 15.

##### Settings governing CES

Three settings restrict how the CES may interact with the external grid:

Setting A (Behind-the-meter coordination). The CES is restricted to internal balancing: charging must originate from surplus PV and discharge may only serve residual community demand:

(Equation 38) $$x_{t}^{\text{imp}}\leq M^{\text{grid}}\left(1-y_{t}^{\text{ch}}\right),$$

(Equation 39) $$x_{t}^{\text{exp}}\leq M^{\text{grid}}\left(1-y_{t}^{\text{dis}}\right).$$

Setting B (Market-enabled procurement). The CES may procure electricity from the grid for charging, but battery discharge may not be exported to the grid:

(Equation 40) $$x_{t}^{\text{exp}}\leq M^{\text{grid}}\left(1-y_{t}^{\text{dis}}\right).$$

Setting C (Fully market-integrated operation). No additional restriction beyond physical battery constraints, Equations 9, 10, 11, 12, and 13. In Setting C, the storage operator faces symmetric buy and sell prices equal to the hourly import price $\pi_{t}^{\text{imp}}$, reflecting full market integration.

##### Community-level optimization objective

(Equation 41) $$\min\;\underset{\text{energy}}{\underset{︸}{\underset{t\in T}{\sum }\left(\pi_{t}^{\text{imp}}\;x_{t}^{\text{imp}}-\pi_{t}^{\text{exp}}\;x_{t}^{\text{exp}}\right)}}+\underset{\text{storage}\;\text{connection}}{\underset{︸}{\underset{t\in T}{\sum }c_{t}^{\text{net}}\;x_{t}^{\text{ch}}-\rho \underset{t\in T}{\sum }x_{t}^{\text{dis}}+\kappa \;{\bar{C}}^{\text{CES}}}}-\underset{\text{network}-\text{fee}\;\text{reduction}}{\underset{︸}{\lambda \;z}},$$

subject to Equations 37 and 21, the applicable institutional constraints, and

(Equation 42) $$z\leq \underset{t\in T}{\sum }x_{t}^{\text{dis,load}},\;z\leq \underset{t\in T}{\sum }x_{t}^{\text{ch,pv}},\;z\geq 0.$$

Household network charges are omitted from the dispatch objective because household metered withdrawals are fixed in Models 3 and 4; these charges are calculated during settlement. By contrast, the shared storage connection cost depends on charging, discharging, and the contracted connection capacity, and is therefore included in the objective.

Here, *z* is the eligible electricity supplied through shared storage, $z=\varphi \sum_{t}x_{t}^{\text{dis,load}}$ in Equation 18, and *λ* is the reduction in community network charges per additional kilowatt-hour of eligible local supply. In Models 3 and 4, the reduction base and the withdrawal $E_{i}^{\text{draw}}$ in Equation 17 are fixed by household load and on-site generation, so $R_{i}^{\text{EC}}$ is linear in $E_{i}^{\text{EC}}$ and the community-average rate is the constant

(Equation 43) $$\lambda =\delta \;\frac{\underset{i\in N}{\sum }\left(\underset{t\in T}{\sum }c_{t}^{\text{net}}x_{i,t}^{\text{draw}}+\kappa \;{\bar{C}}_{i}+12f\right)}{\underset{i\in N}{\sum }E_{i}^{\text{draw}}},$$

which depends only on the tariff parameters and on the exogenous household withdrawals, not on any dispatch decision. Because the reduction enters the objective as a benefit, the constraints in Equation 42 implement the eligibility cap *φ* without binary variables. Dispatch uses the community-average rate, whereas settlement calculates each household’s reduction using Equation 17. Including this incentive in dispatch captures the value of eligible local supply that would otherwise be overlooked if the reduction were applied only after optimization.

In Model 3, dispatch is coordinated on behalf of community members. In Model 4, the same community-level dispatch objective is assumed for the third-party operator, allowing ownership effects on value capture to be compared under a common operating objective.

##### Ex-post flow decomposition (both models)

After optimization, realized energy flows are attributed using the following priority-based accounting rule, applied identically in Models 3 and 4.

Step 1: Direct local PV sharing.

(Equation 44) $$x_{t}^{\text{pv,loc}}=\min\;\left(D_{t}^{\text{res}},\;P_{t}^{\text{sur}}\right),$$

(Equation 45) $$D_{t}^{\text{gap}}=D_{t}^{\text{res}}-x_{t}^{\text{pv,loc}},$$

(Equation 46) $$P_{t}^{\text{excess}}=P_{t}^{\text{sur}}-x_{t}^{\text{pv,loc}}.$$

Step 2: CES charging decomposition.

(Equation 47) $$x_{t}^{\text{ch,pv}}=\min\;\left(x_{t}^{\text{ch}},\;P_{t}^{\text{excess}}\right),$$

(Equation 48) $$x_{t}^{\text{imp,bat}}=x_{t}^{\text{ch}}-x_{t}^{\text{ch,pv}},$$

(Equation 49) $$x_{t}^{\text{imp,load}}=x_{t}^{\text{imp}}-x_{t}^{\text{imp,bat}}.$$

Step 3: CES discharging decomposition.

(Equation 50) $$x_{t}^{\text{dis,load}}=\min\;\left(x_{t}^{\text{dis}},\;D_{t}^{\text{gap}}\right),$$

(Equation 51) $$x_{t}^{\text{exp,bat}}=x_{t}^{\text{dis}}-x_{t}^{\text{dis,load}},$$

(Equation 52) $$x_{t}^{\text{exp,pv}}=x_{t}^{\text{exp}}-x_{t}^{\text{exp,bat}}.$$

##### Household-level allocation of decomposed flows

(Equation 53) $$x_{i,t}^{\text{buy,loc}}=\frac{{\tilde{d}}_{i,t}^{\text{res}}}{D_{t}^{\text{res}}}\;x_{t}^{\text{pv,loc}},x_{i,t}^{\text{sell,loc}}=\frac{{\tilde{p}}_{i,t}^{\text{sur}}}{P_{t}^{\text{sur}}}\;x_{t}^{\text{pv,loc}},$$

(Equation 54) $$x_{i,t}^{\text{dis,load}}=\frac{{\tilde{d}}_{i,t}^{\text{res}}}{D_{t}^{\text{res}}}\;x_{t}^{\text{dis,load}},x_{i,t}^{\text{ch,pv}}=\frac{{\tilde{p}}_{i,t}^{\text{sur}}}{P_{t}^{\text{sur}}}\;x_{t}^{\text{ch,pv}},$$

(Equation 55) $$x_{i,t}^{\text{imp,load}}=\frac{{\tilde{d}}_{i,t}^{\text{res}}}{D_{t}^{\text{res}}}\;x_{t}^{\text{imp,load}},x_{i,t}^{\text{exp,pv}}=\frac{{\tilde{p}}_{i,t}^{\text{sur}}}{P_{t}^{\text{sur}}}\;x_{t}^{\text{exp,pv}}.$$

All ratios are set to zero when the denominator equals zero.

##### Model 3: Community-owned storage

Model 3 represents a collectively governed energy community in which households jointly own and operate the CES. Ownership shares *w*_*i*_ ≥ 0 satisfy $\sum_{i\in N}w_{i}=1$ but do not influence dispatch; they govern only ex-post redistribution of storage-related value, thereby separating operational efficiency from distributive outcomes.

The physical model, institutional settings, optimization objective, and flow decomposition are as specified under Community energy storage: Models 3 and 4.

##### Ex-post cost allocation

*Grid-related costs* are allocated using only the load-serving components:

(Equation 56) $$C_{i}^{\text{grid}}=\underset{t\in T}{\sum }\left(\pi_{t}^{\text{imp}}\;x_{i,t}^{\text{imp,load}}-\pi_{t}^{\text{exp}}\;x_{i,t}^{\text{exp,pv}}\right).$$

Storage usage charge and PV-charging credit:

(Equation 57) $$U_{i}^{\text{bat}}=\underset{t\in T}{\sum }\pi_{t}^{\text{loc}}\;x_{i,t}^{\text{dis,load}},$$

(Equation 58) $$R_{i}^{\text{bat}}=\underset{t\in T}{\sum }\pi_{t}^{\text{loc}}\;x_{i,t}^{\text{ch,pv}}.$$

Local sharing cost and revenue:

(Equation 59) $$C_{i}^{\text{buy,loc}}=\underset{t\in T}{\sum }\pi_{t}^{\text{loc}}\;x_{i,t}^{\text{buy,loc}},$$

(Equation 60) $$R_{i}^{\text{sell,loc}}=\underset{t\in T}{\sum }\pi_{t}^{\text{loc}}\;x_{i,t}^{\text{sell,loc}}.$$

##### CES settlement account

All storage-related revenues and costs are pooled, including the network charge on the storage connection (Equation 20), which under community ownership the community itself bears:

(Equation 61) $$\Pi^{\text{CES}}=\underset{i\in N}{\sum }U_{i}^{\text{bat}}+\underset{t\in T}{\sum }\pi_{t}^{\text{exp}}\;x_{t}^{\text{exp,bat}}-\underset{i\in N}{\sum }R_{i}^{\text{bat}}-\underset{t\in T}{\sum }\pi_{t}^{\text{imp}}\;x_{t}^{\text{imp,bat}}-N^{\text{CES}}.$$

The net storage value is redistributed to households according to ownership shares:

(Equation 62) $$O_{i}^{\text{own}}=w_{i}\;\Pi^{\text{CES}}.$$

##### Household electricity cost

(Equation 63) $$C_{i}^{\text{M}3}=C_{i}^{\text{grid}}+U_{i}^{\text{bat}}+C_{i}^{\text{buy,loc}}-R_{i}^{\text{sell,loc}}-R_{i}^{\text{bat}}-O_{i}^{\text{own}}+N_{i}^{\text{net}}.$$

##### Model 4: Third-party-owned storage

Model 4 represents an institutional configuration in which the CES is owned and operated by an independent third-party storage service provider. The third party aggregates surplus PV generation, facilitates local energy balancing, and optimizes storage dispatch with respect to external electricity markets, but does not act as a licensed electricity retailer.

The physical model, institutional settings (Settings A–C), optimization objective, and flow decomposition are identical to those specified under Community energy storage: Models 3 and 4.

##### Ex-post settlement: household cost

The storage service fee *τ* ≥ 0 (per kWh discharged to load) and the fixed CES participation fee *F*^CES^ are levied by the third party. The fixed cost share of household *i* is *α*_*i*_, with $\sum_{i\in N}\alpha_{i}=1$. The per-unit storage service fee *τ* = 0.02 CHF/kWh reflects a marginal service charge on battery-mediated energy delivery.

##### Household electricity cost

(Equation 64) $$\begin{array}{cc} C_{i}^{\text{M}4}=\underset{t\in T}{\sum } & \left[\pi_{t}^{\text{loc}}\;x_{i,t}^{\text{buy,loc}}+\left(\pi_{t}^{\text{loc}}+\tau \right)\;x_{i,t}^{\text{dis,load}}+\pi_{t}^{\text{imp}}\;x_{i,t}^{\text{imp,load}}-\pi_{t}^{\text{loc}}\;x_{i,t}^{\text{sell,loc}}-\pi_{t}^{\text{loc}}\;x_{i,t}^{\text{ch,pv}}-\pi_{t}^{\text{exp}}\;x_{i,t}^{\text{exp,pv}}\right]\\ & +\alpha_{i}\;F^{\text{CES}}+N_{i}^{\text{net}}. \end{array}$$

##### Third-party revenue

Under third-party ownership the operator holds the storage connection and bears its network charge (Equation 20), whereas under community ownership that charge falls on the community (Equation 61). This is the only difference in the network treatment of the two ownership arrangements, and it is what makes third-party ownership preferable to the community wherever the arbitrage the operator extracts is worth less than the connection charge it absorbs.

(Equation 65) $$R^{3\text{rd}}=\underset{t\in T}{\sum }\left[\left(\pi_{t}^{\text{loc}}+\tau \right)\;x_{t}^{\text{dis,load}}-\pi_{t}^{\text{loc}}\;x_{t}^{\text{ch,pv}}+\pi_{t}^{\text{exp}}\;x_{t}^{\text{exp,bat}}-\pi_{t}^{\text{imp}}\;x_{t}^{\text{imp,bat}}\right]+F^{\text{CES}}-N^{\text{CES}}.$$

##### Model assumptions

The analysis is based on a set of simplifying assumptions to enable a consistent comparison across institutional configurations. Battery storage capacity is defined relative to average daily demand, providing a simple, demand-based reference for accommodating daily variations in PV generation and consumption. This sizing rule also enables consistent comparisons across communities of different sizes. Battery charging and discharging efficiencies are assumed constant (*η*^ch^ = *η*^dis^ = 0.95). Perfect foresight of demand, generation, and price signals is assumed in the dispatch optimization. Network constraints are not explicitly modeled, and electricity prices and network tariffs are treated as exogenous inputs under each pricing regime and tariff archetype (see prices, tariffs and regulatory parameters below). The system configuration, including the number of households and their load and generation profiles, is fixed throughout the analysis. These assumptions allow the study to isolate the effects of institutional design on value creation, distribution, and system interaction.

##### Model implementation and solver

The models are implemented in Python 3.13. Models 2–4 are formulated as hourly mixed-integer linear programs and solved with Gurobi 13.0.1. Binary variables prevent simultaneous charge and discharge and, where relevant, simultaneous import and export or prohibited market actions. The relative MIP optimality tolerance is 10^–3^. A deterministic work limit is used for reproducibility; where the limit binds, the best incumbent is retained and the achieved optimality gap recorded. Across all reported runs, the tolerance is met except for a small number of capacity-charge cases under dynamic pricing, for which the final gap remains below 0.3%.

##### Prices, tariffs and regulatory parameters

This section summarizes the price, tariff, regulatory, and robustness assumptions used in the analysis. Unless otherwise stated, monetary values are reported in Swiss francs (CHF) excluding VAT.

##### Currency and value-added tax

Swiss VAT is 8.1%. Because the same VAT rate applies to grid electricity, community electricity, network charges, and the statutory network-charge reduction, VAT scales both costs and savings proportionally and does not affect the relative indicators reported in this study. Amounts published only on a VAT-inclusive basis are divided by 1.081. Euro-denominated values are converted using the 2025 annual average exchange rate of 1 EUR = 0.9370 CHF.

##### Supply context of the case study

The case study is located in the Glattwerk AG distribution area in Dübendorf, Switzerland. All seven units are connected at grid level 7. Since 1 January 2026, Swiss regulation permits pLEG, *lokale Elektrizitätsgemeinschaften* in Swiss law, in which participants retain individual metering points and exchange locally generated electricity over the public distribution grid at a reduced network usage tariff.^76^ The seven units are modeled as separate metering points forming one LEG.

##### Electricity price regimes

The three retail price regimes describe only the *energy* component of the electricity price; network charges are varied independently under network tariff archetypes below.

The ToU regime follows the 2026 Glattwerk household tariff,^83^ see Table 3. The high-tariff period is Monday–Friday 07:00–20:00 and Saturday 07:00–13:00; all remaining hours are low tariff. Summer runs from 1 April to 30 September and winter covers the remaining months.

The flat regime is defined as the load-weighted annual mean of the ToU tariff, equal to 12.29 Rp/kWh (1 Rp = 0.01 CHF) for the case study community. The dynamic regime uses 2025 Swiss day-ahead market prices from the ENTSO-E Transparency Platform,^84^ converted to CHF and shifted by a constant supplier markup so that its load-weighted annual mean also equals 12.29 Rp/kWh. The raw day-ahead series has a load-weighted annual mean of 10.36 cEUR/kWh, corresponding to 9.71 Rp/kWh after currency conversion; the resulting markup is 2.59 Rp/kWh. Equalizing the annual mean across regimes isolates the effect of temporal price variation.

##### Network tariff archetypes

Network tariffs are represented through three archetypes that distinguish whether network costs are recovered from electricity withdrawal, contracted connection capacity, or both, together with an energy-only benchmark, as shown in Table 4. T0 is an energy-only benchmark without network charge. T1 applies a volumetric charge, T2 a charge on contracted connection capacity, and T3 combines the two. The three network tariff archetypes are calibrated *ex ante* to recover the same annual network revenue from the reference configuration (Model 0), CHF 3697.6 across the seven household connections. This revenue-neutral calibration isolates the effect of the tariff structure from differences in the initial level of network cost recovery. Because operation subsequently responds to the tariff, realized network revenues need not remain identical across configurations.

T1 corresponds to the standard low-voltage household tariff applied at the case study location.^83^ T2 represents network-cost recovery through contracted connection capacity, following the structure used for Dutch small consumer connections.^87^ T3 combines both charging bases. Its volumetric–capacity split is derived from the published Glattwerk business tariff,^85^ but its overall level is rescaled to the same reference case revenue as T1 and T2. Fixed and metering charges are kept identical across the three network tariff archetypes. Detailed tariff parameters are reported in Table 5.

Network charges are assessed at individual metering points. A household’s metered withdrawal is its electricity demand net of on-site PV self-consumption. Electricity received from another community member or discharged from shared storage passes through the distribution network and is withdrawn at the receiving household meter; it therefore remains subject to the standard network tariff before the statutory reduction for eligible local electricity is applied (see network tariff reduction for local supply below). Consequently, local energy sharing and shared storage do not directly reduce the household’s gross metered withdrawal. In contrast, storage located behind an individual household meter can reduce volumetric network charges by reducing withdrawal at that meter. Under a pure contracted-capacity tariff, this benefit arises only if the household is able to reduce its contracted connection capacity.

The shared storage system is represented as an additional metering point with its own network connection. Under T2 and T3, the storage connection is selected from a discrete set of standard three-phase connection ratings, from 3 × 25 A to 3 × 80 A (17.3–55.4 kW), and is optimized jointly with storage operation. The contracted connection limits both charging and discharging power, creating a trade-off between the cost of connection capacity and the amount of installed storage power that can be used. Under T0 and T1, connection capacity is not priced and the connection is assumed sufficient for the installed storage power.

For the storage connection, volumetric network charges apply to electricity withdrawn for charging. All electricity discharged from the storage, either fed back into the distribution grid or supplied to the local community, is eligible for reimbursement of the volumetric network usage charge under the published storage tariff^83^; the reimbursement prevents the same kilowatt-hour from being charged at the storage connection and again at the consuming connection. The reimbursement is applied at a single rate *ρ* per kilowatt-hour discharged, set equal to the load-weighted annual mean of the volumetric charge in the reference case: 7.50 Rp/kWh under T1 and 3.32 Rp/kWh under T3, and zero under T2, which has no volumetric component. A single rate is used because the published clause consolidates the refundable components into one tariff whose value is not published; reimbursing at the high- and low-tariff rates of the discharge hour would instead allow storage to earn 1.0 Rp/kWh from the tariff calendar alone by charging in low-tariff and discharging in high-tariff hours. The resulting volumetric network cost therefore depends mainly on round-trip losses and on differences between charging and discharging tariff periods. Contracted capacity charges are not refundable and act directly on the connection capacity required by the storage system. This distinction is central to the comparison between volumetric and capacity-based network tariffs.

Taxes, levies, and other statutory charges that are not part of network-cost recovery are excluded from the network tariff archetypes. The statutory reduction granted to electricity supplied within an energy community is described separately under network tariff reduction for local supply.

##### Network tariff reduction for local supply

Swiss law grants energy community participants a reduction on the network usage tariff for electricity generated and consumed within the community. Article 19h of the StromVV sets the reduction at 40% when no voltage transformation is required between generation and consumption, and 20% otherwise.^76^ Because all case study participants are connected at the same low-voltage level, the 40% rate is used in the baseline. Sensitivity results are reported for *δ* ∈ {0, 0.20, 0.40, 0.60, 1.00}, spanning a broad regulatory range. Dispatch is held at the schedule optimized for the baseline value to isolate the direct effect of changing the reduction rate.

##### Feed-in remuneration

Surplus PV electricity exported to the grid is remunerated at 0.06 CHF/kWh, corresponding to the statutory minimum remuneration applicable to the case study PV systems.^86^

##### Foresight and rolling-horizon test

The main optimization is solved over the full year with perfect foresight. Robustness is assessed using rolling-horizon operation with 24 h commitment. Two look-ahead windows are considered: 24 h, with no look-ahead beyond the committed day, and 48 h, providing an additional day of look-ahead. The storage SoC is carried between windows, and contracted connection capacity is fixed at the annual value.

Window *k* covers hours *t*_*k*_, …, *t*_*k*_ + *W* − 1, with *t*_*k*_ = 24(*k* − 1) and *W* ∈ {24, 48} h. In each window, the dispatch problem of Models 3 and 4 is solved with the SoC initialized at the value reached at the end of the previously committed day, $s_{t_{k}}=s_{t_{k-1}+24}^{*}$. No terminal SoC constraint is imposed within individual rolling windows. Only the first 24 h of dispatch are implemented, and the resulting SoC is carried forward to the next window.

This allows energy to be retained across daily boundaries without requiring the battery to return to a prescribed SoC after each committed day. The 48 h window provides an additional day of look-ahead compared with the 24 h window, although both formulations remain subject to finite-horizon effects. Demand, PV generation, and prices are assumed to be known over each optimization window. The committed annual trajectory is evaluated on the full community cost, settled on the realized flows as in Model 3: energy purchases net of export revenue, household network charges net of the statutory reduction, and the storage connection charge net of its reimbursement.

With 48 h look-ahead, the increase in annual community cost is at most 1.65% and at most 0.81% for full market access. With 24 h look-ahead the increase reaches 14.8% for import-only access under T0 and 5.9–6.9% under the charged archetypes. The import-only configuration is more sensitive to the information horizon because its value depends more strongly on carrying energy across daily boundaries. Demand and PV forecast errors are not modeled in this study.

### Quantification and statistical analysis

This paper does not contain any statistical analysis.
